# Supplementary material for: Characterizing the particulate content of urine in healthy humans using flow cytometry
Source: PLoS One. 2025 May 22;20(5):e0324271. doi: 10.1371/journal.pone.0324271 (PMC12097575; doi:10.1371/journal.pone.0324271)
Supplement: S1 File — S.1 – S.9, that include information on methods, detailed data and results, and statistical analysis. S1 Table. Commercial fluorescence tags that were used to label particulate matter in urine. S2 Table. Laser setting for the experiments in the IFC. S3 Table. Description of the channels and their recorded target in the IFC. S4 Table. Mean ± STD and range (minimum, maximum values in parentheses) particle concentration results for different collection vessels at different preparation times for 3 samples of a single donor. FIM: first-in-morning sample with preparation 3 hours post collection, LP: late-preparation 7 hours post collection, FIM + LP: daily average. S5 Table. Mean ± STD and range (minimum, maximum values in parentheses) particle mean area results for different collection vessels at different preparation times for single donors. FIM: first-in-morning sample with preparation 3 hours post collection, LP: late-preparation 7 hours post collection, FIM + LP: daily average. S6 Table. Mean ± STD and range (minimum, maximum values in parentheses) results of particle concentration for different times for 18 donors individually. FIM: first-in-morning sample, LM: late-morning samples, FIM + LM: daily average. S7 Table. Mean ± STD and range (minimum, maximum values in parentheses) results of particle mean area for different times for 18 donors individually. FIM: first-in-morning sample, LM: late-morning samples, FIM + LM: daily average. S8 Table. Mean ± SE and range (minimum, maximum values in parentheses) results for different times for 18 donors. FIM: first-in-morning sample, LM: late-morning samples, FIM + LM: daily average. S9 Table. Mean ± SE and range (minimum, maximum values in parentheses) results of particle concentration for different times for two gender groups, each containing 9 donors. FIM: first-in-morning sample, LM: late-morning samples, FIM + LM: daily average. S10 Table. Mean ± SE and range (minimum, maximum values in parentheses) results of particle m [file pone.0324271.s001.docx]

**SUPPORTING INFORMATION**

**Characterizing the particulate content of urine in healthy humans using flow cytometry**

Sigal Hirsch^1^, Ziv Porat^2^, Ishai Dror^1^, Yaniv Shilo^3^, and Brian Berkowitz^1*^

^1^Department of Earth and Planetary Sciences, Weizmann Institute of Science, Rehovot 7610001, Israel email: [sigal.hirsch@gmail.com](mailto:sigal.hirsch@gmail.com), [ishai.dror@weizmann.ac.il](mailto:ishai.dror@weizmann.ac.il)

^2^Life Sciences Core Facilities, Weizmann Institute of Science, Rehovot 7610001, Israel email: [ziv.porat@weizmann.ac.il](mailto:ziv.porat@weizmann.ac.il)

^3^Department of Urology, Kaplan Medical Center, Affiliated with the Hebrew University, Rehovot 7661041 **Israel** email: [drshiloy@gmail.com](mailto:drshiloy@gmail.com)

*Corresponding author: Brian Berkowitz, Department of Earth and Planetary Sciences, Weizmann Institute of Science, Rehovot 7610001, Israel email: [brian.berkowitz@weizmann.ac.il](mailto:brian.berkowitz@weizmann.ac.il)

**Contents:**

S.1 Methods

S.2 Sample Storage, Preparation Times, and Result Variability

S.3 Overall Results – Total Particles

S.4 Overall Results Grouped Data by Sample Time

S.5 Overall Results Grouped Data by Gender and Sample Time

S.6 Overall Results Grouped Data by Age Group and Sample Time

S.7 Overall Results Grouped Data by Gender and Age Group

S.8 Statistical Analysis

## S.9 Overall Results – Crystals

# S.1 Methods

S.1.1 Birefringence

Imaging flow cytometry (IFC) is a powerful analytical technique that allows for the measurement of various bright-field and fluorescence parameters, providing detailed information on particle staining intensity, distribution patterns, localization, and morphological features.

Beyond fluorescent tagging, the ability to analyze crystalline structures by birefringence was recently added to IFC (Shaked et al., manuscript in preparation). The ImageStreamX was fitted with a custom-made setup, integrating perpendicular polarizers positioned near the light source and the detector. This allowed the system to detect birefringent crystals, i.e. calcium oxalate or other mineral deposits associated with kidney stones, as well as other birefringence material such as proteins and lipid droplets. This setup enhances the ability to visualize and quantify crystalline particles, which are often missed or underestimated in traditional urine analysis methods. To compensate for light loss, the LED power was manually increased to 95 mW.

## S.1.2 Labeling

The tags are designed to label particles containing, individually or collectively, lipids, protein aggregates, calcium, and DNA (i.e. cells and bacteria), as detailed in Table S1. Particles containing multiple components can be labeled with multiple fluorescent tags.

Table S1. Commercial fluorescence tags that were used to label particulate matter in urine.

| **Name** | **Specific Labeling** | **Concentration** | **Incubation time [min]** | **Supplier** | **Product name** | **Catalog number** |
| --- | --- | --- | --- | --- | --- | --- |
| LipidTox | Neutral lipids | 1:200 in DDW | 40 | Thermo Fisher Scientific | HCS LipidTOX™ Deep Red | H34477 |
| Proteostat | Peptide and protein aggregation in solution | 1:1000 in buffer | 10 | Enzo | PROTEOSTAT® Protein aggregation assay | ENZ-51023-KP002 |
| Calcein | Calcium | 0.625 mg/mL | 40 | Sigma-Aldrich | Calcein | C0875-5G |
| Hoechst | Double stranded DNA | 1 mg/mL | 40 | Thermo Fisher Scientific | Hoechst 33342, Trihydrochloride, Trihydrate | H3570 |

## S.1.3 Flow Cytometry

Each sample was imaged and analyzed using an IFC (ImageStreamX Mark II, Amnis-part of Cytek Biosciences, CA, USA). Lasers were set to excite the fluorescent tags (Table S2). Each fluorescent tag has a unique emission wavelength, recorded on separate channels in the flow cytometer (Table S3).

Table S2. Laser setting for the experiments in the IFC.

| **Laser wavelength [nm]** | 405 | 488 | 561 | 642 | 785 |
| --- | --- | --- | --- | --- | --- |
| **Intensity [mW]** | 120 | 200 | 200 | 150 | 1 |

Table S3. Description of the channels and their recorded target in the IFC.

| **Channel** | **Bandwidth [nm]** | **Measurement** | **Dye Excitation/Emission [nm]** | **Target** |
| --- | --- | --- | --- | --- |
| 1 | 430-480 | Polarized Light | - | Birefringence |
| 2 | 480-560 | Calcein | 470/509 | Particles containing calcium |
| 4 | 595-640 | Proteostat | 500/610 | Protein Aggregates |
| 6 | 745-800 | Side Scatter | - | - |
| 7 | 430-385 | Hoechst | 350/461 | DNA |
| 9 | 570-595 | Bright Field | - | - |
| 11 | 640-745 | LipidTox | 635/650 | Neutral Lipids |

##

## S.1.4 Analysis and Initial Testing

### *Identification of Cells:* First, cells were identified using a bivariate plot of intensity (total fluorescence emitted by an object) versus max pixel (brightness of the most intense pixel) in the Hoechst channel. Particles with high intensity and max pixel values were classified as cells or cell fragments, while lower values indicated non-cells. Figure S2a, below, illustrates the classification approach used to distinguish between cells and other particles.

### *Identification of Bacteria:* All samples from females exhibited bacteria; few to no bacteria were detected in the samples from males. The bacteria are illustrated in Figure S1.


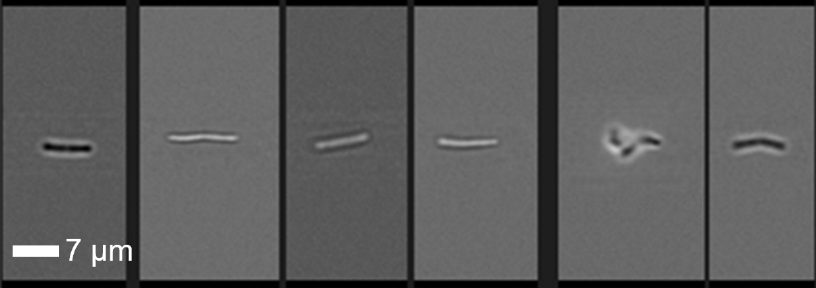


Figure S1. Typical bacteria found in samples from females.

Figure S2 demonstrates a clear distinction between samples from males and females, showing a noticeable bacterial population in the samples from females. However, because both bacteria and cells were tagged with Hoechst and exhibited similar intensities, it was not possible to separate them into distinct populations for more detailed analysis.


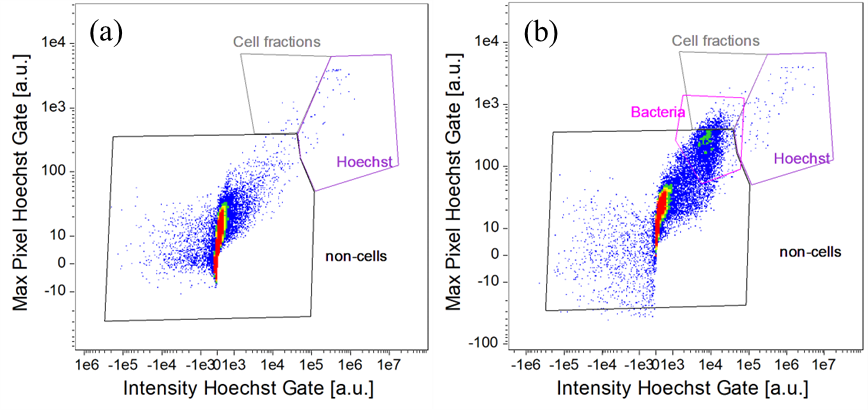


Figure S2. Population density scatter graphs of all measured objects in IFC, intensity vs. max pixel of Hoechst of (a) male, and (b) female volunteers. High values of both indicate the object measured is a cell. The bacteria population was marked in the sample from a female.

## *Identification of Non-Cell Particles:* The subsequent analysis focused on non-cell particles. For each fluorescent tag, a bivariate plot of the max pixel of the specific tag (the highest intensity pixel value within the image mask) was compared to the sum of the max pixel values of all other tags, to ensure that the positive fluorescent signal emanates from the particular channel for each dye and does not reflect mixed staining. For example, the “All but Calcein” parameter was calculated as: Max Pixel_M04_Proteostat + Max Pixel_M07_Hoechst + Max Pixel_M11_LipidTox.

### This analysis allowed for the differentiation between particles tagged with one fluorescent marker, multiple markers, or no markers. The gating was determined according to the single stain controls and intrinsic fluorescence. Figure S3 demonstrates this process with Calcein, LipidTox, and Proteostat tags, revealing distinct populations.


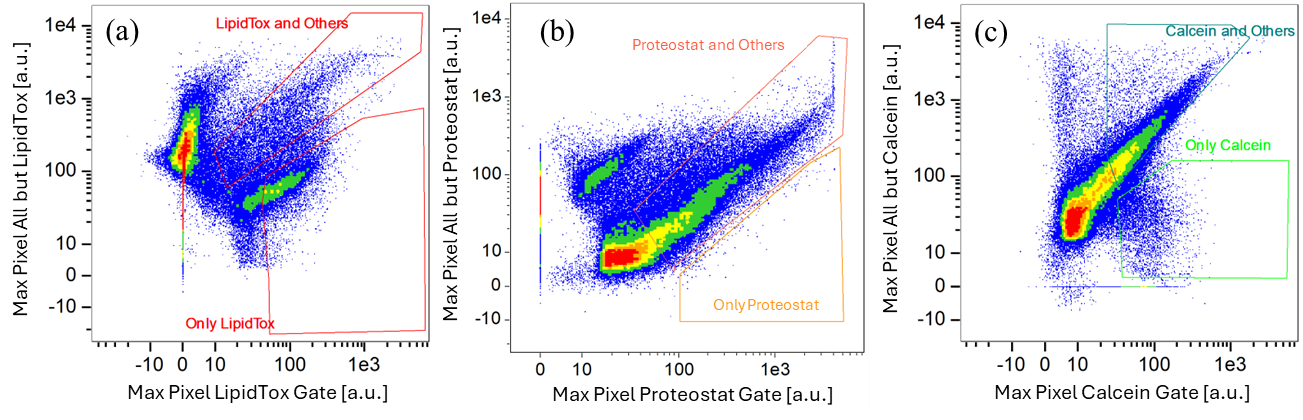


Figure S3. Population density scatter graph of the measured objects in IFC of max pixel fluorescent tag of (a) LipidTox, (b) Proteostat, and (c) Calcein vs. sum of max pixel values of all other tags, taken from the “non-cells” population. Apart from Proteostat, 3 populations can be seen: only the tag, the tag, and other tags, and no tag. For Protestat, only 2 populations exist: Proteostat and LipidTox plus others.

Analysis of Proteostat-stained particles indicated the absence of a single-stained population; instead, it indicated a mixed population with Proteostat and LipidTox tagging. A scatter plot of max pixel Proteostat vs. max pixel LipidTox identified three populations: those tagged predominantly with Proteostat, those with LipidTox, and those with a mixture of both, as shown in Figure S4. This population was established as a relevant population for further study and named “Lipid-Proteins”, an aggregation of both proteins and lipids.


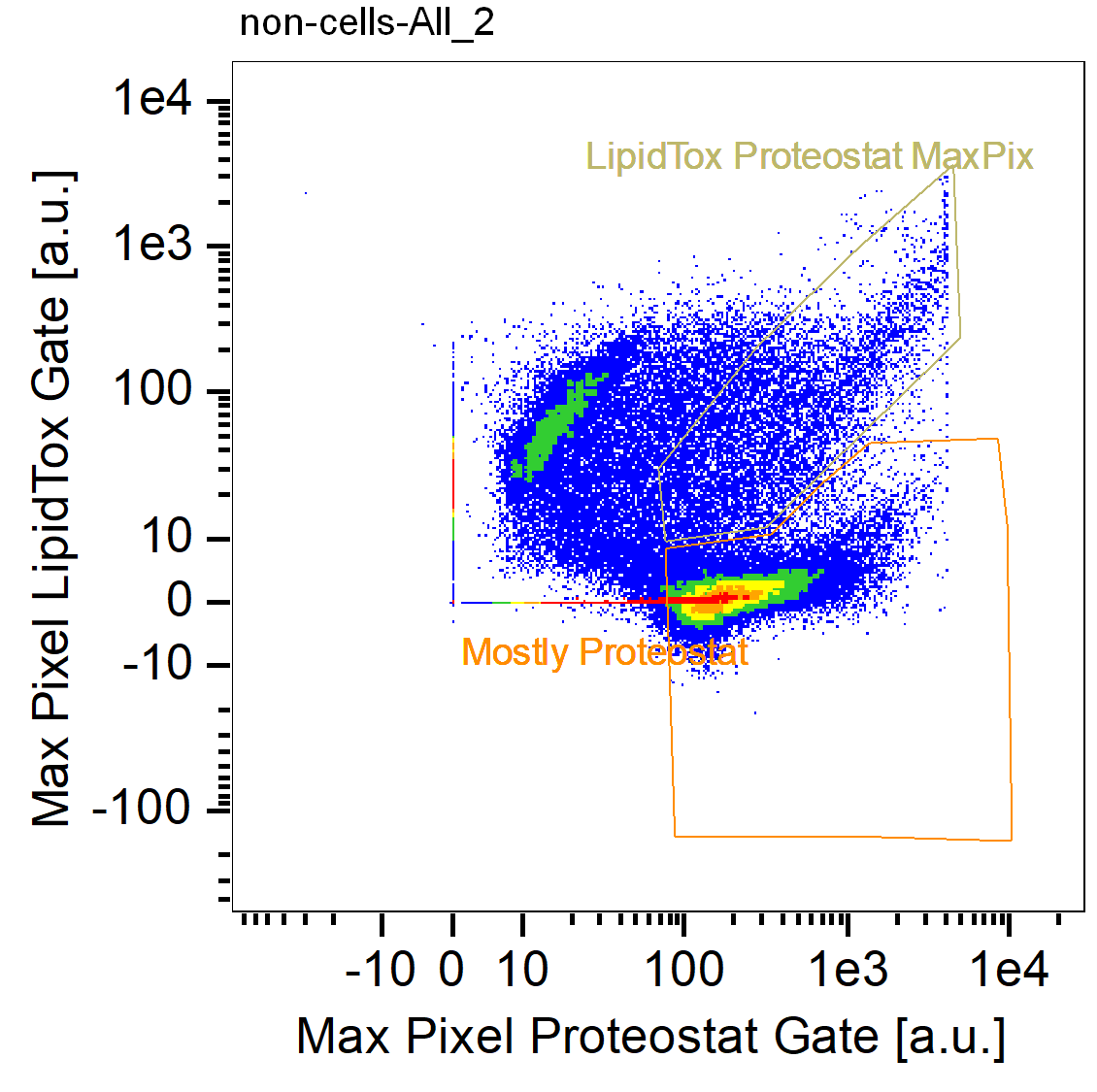


Figure S4. Density population of max pixel Protestat vs. max pixel LipidTox, taken from the “non-cells” population. Three populations are shown: Proteostat tagged, LipidTox tagged, and a mix.

###

### *Crystal Analysis and Estimation Methods:* The IFC analysis of crystals in urine – detecting particles in the polarized channel – is not straightforward due to the potential variability in the form and composition of particles containing calcium, which do not always bind reliably to the Calcein dye. As a result, Calcein alone proved to be an unreliable marker for detecting crystals, necessitating the development of alternative methods. Three approaches were evaluated to estimate crystal content in urine samples:

- Method 1: Directly comparing object size and max pixel intensity in the birefringence channel, with stronger signals assumed to indicate crystals. Named “Birefringence by Area”.
- Method 2: Comparing the max pixel values of the birefringence signal with Calcein, based on the assumption that crystals contain calcium. Named “Birefringence by Calcein”.
- Method 3: Cross-referencing birefringence signals against all non-Calcein signals, under the assumption that crystals should not bind to proteins, lipids, or DNA. Named “Only Birefringence”.

These methods are illustrated in Figure S5, showing the different approaches to the identification of crystals in the samples.


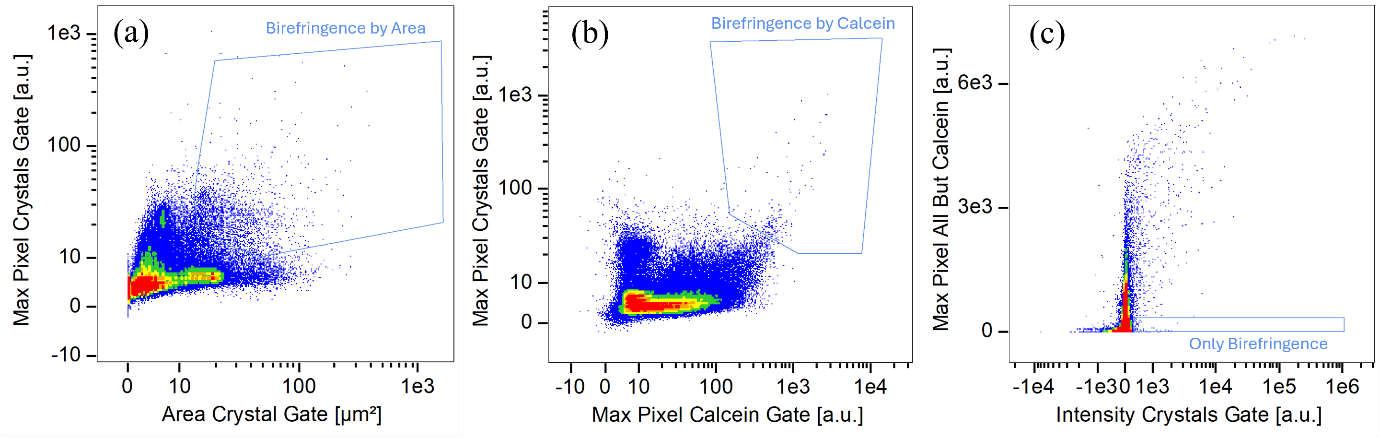


Figure S5. Three methods of finding crystals (taken from the “non-cells” population): (a) directly comparing the birefringence channel max pixel to the object size and taking the higher values of both, (b) comparing the birefringence channel max pixel to Calcein max pixel and taking the higher values of both and (c) comparing the birefringence channel max pixel to all tags but Calcein max pixel and taking the maximum of birefringence and minimum of the tags.

Figure S6 shows the distribution of particle sizes of each of the methods for the 108 samples, while Figure S7 displays the results of these methods across all participants, including the outlier. Among the 19 volunteers, only three (coded Female 9 (F9), Male 7 (M7), and Outlier 1 (O1)) exhibited any crystal-like behavior, and even then, only in some of their samples. In total, fewer than 10 of the 108 samples showed a significant presence of crystals, resulting in an overall crystal identification rate of approximately 8%, regardless of whether or not the identified particles were indeed crystals. The following sections focus on analyzing the identified particles in the relevant crystal classifications for O1, F9, and M7. Full data are available in Section S.9.


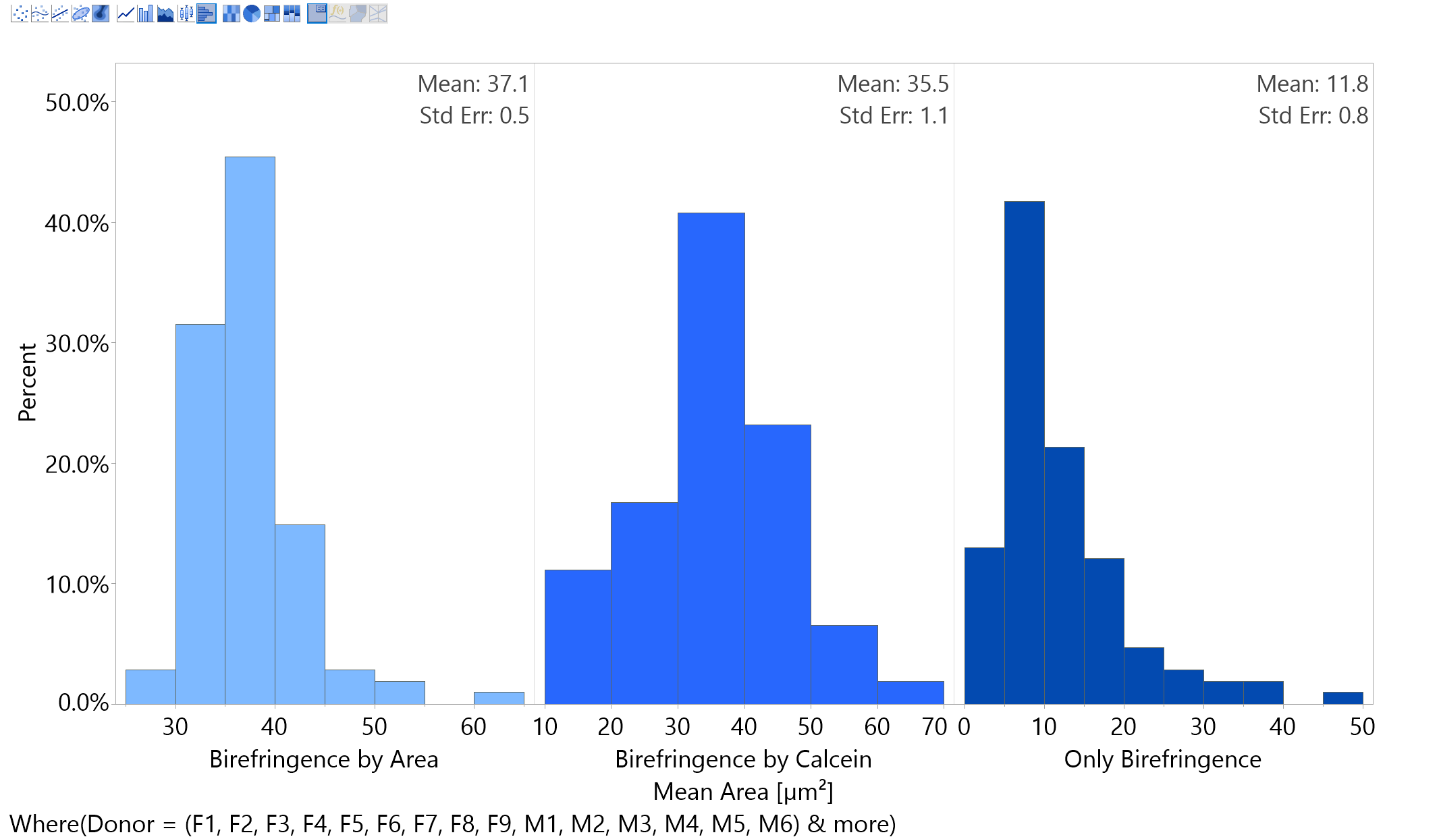


Figure S6. Histogram distribution of mean area of particulate content of 108 samples from the populations of three crystal classification. The mean and std of each histogram appear in the top right of each block.


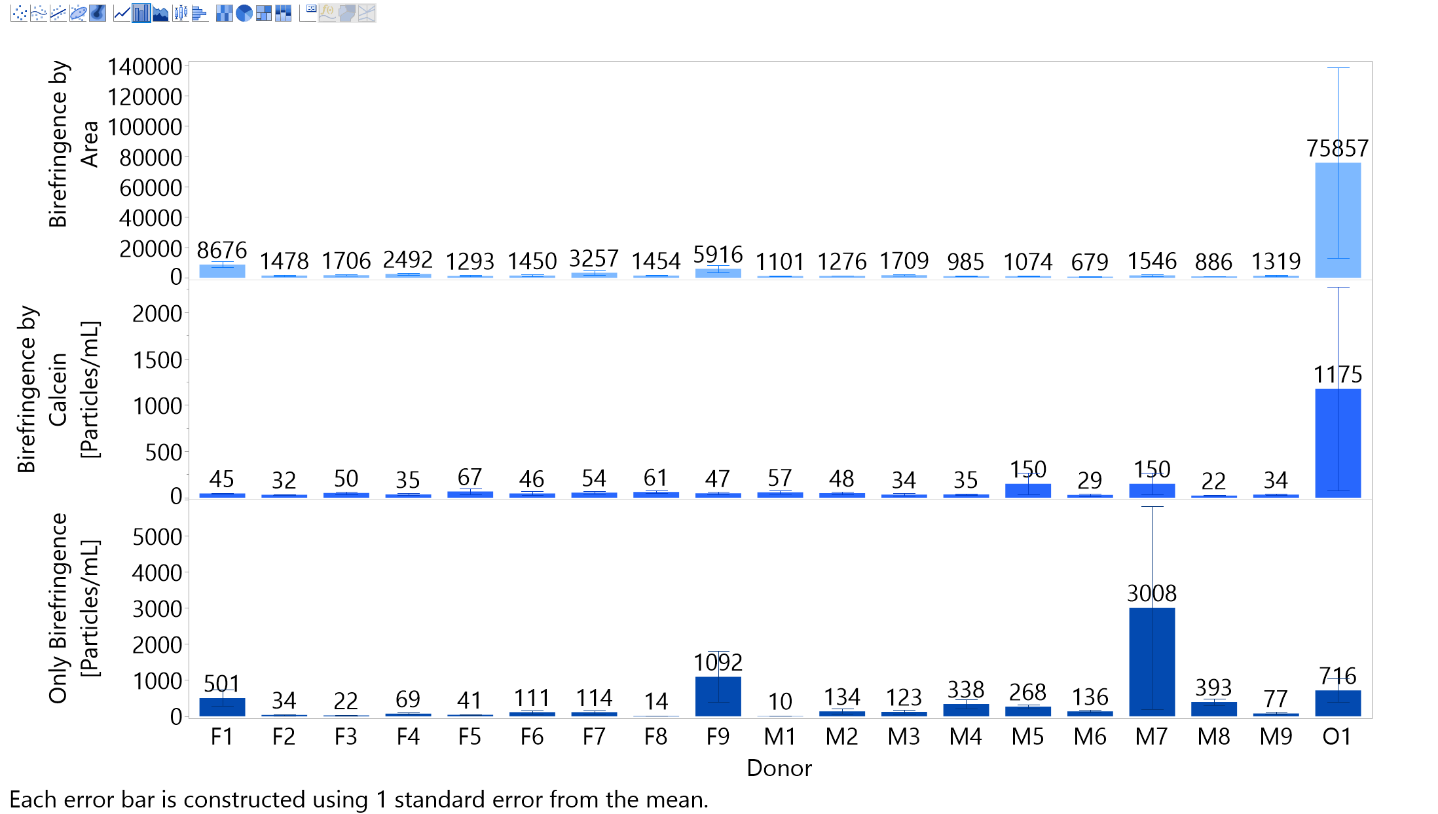


Figure S7. Comparative analysis of particulate matter concentrations in urine samples of 19 participants. The three rows represent different methods of crystal estimation. Each error bar represents one standard error from the mean. F = Female volunteer, M = Male volunteer, O = Outlier, numbered by ascending age.

### Method 1: Birefringence by Area

The "Birefringence by Area" population was defined by comparing particle area, as measured automatically by the imaging flow cytometer (IFC), to the max pixel intensity in the birefringence detection channel, with higher values considered part of the population. Under this definition, only participant O1 showed a notable concentration, with other volunteers maintaining a consistent daily concentration of approximately 1500 particles/mL. Figure S8 presents a scatter graph of this population where it was clear that the O1 sample was significantly more concentrated within the population limits.


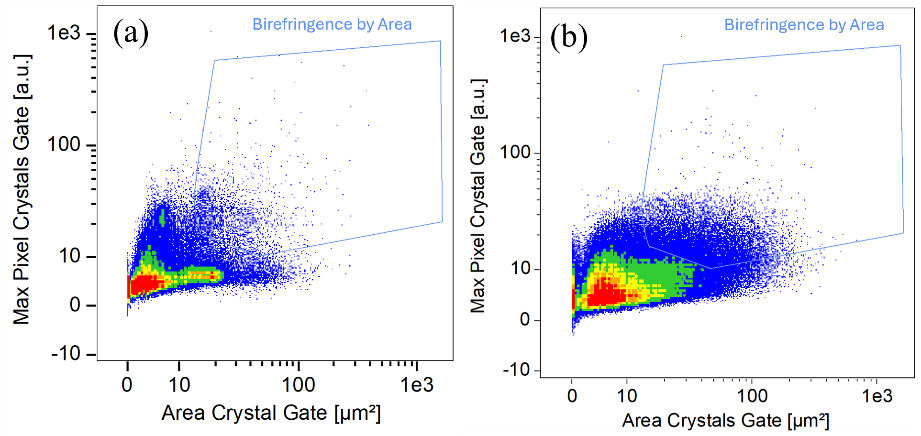


Figure S8. Population density scatter graphs of the measured objects in IFC, area vs. max pixel of particles in the birefringence gate of (a) typical sample and (b) O1 sample, taken from the “non-cells” population. Birefringence by Area population is marked on the figures.

The particles identified under this definition in the O1 sample are shown in Figure S9. In addition to the birefringence signal, these particles displayed strong LipidTox and Proteostat signals, with occasional Calcein tagging. The particles were not pure in the stream, as bacteria can be seen beside the particles that gave birefringence signals. It was unclear whether the detected particles were actual crystalline structures or Lipid-Proteins aggregates.


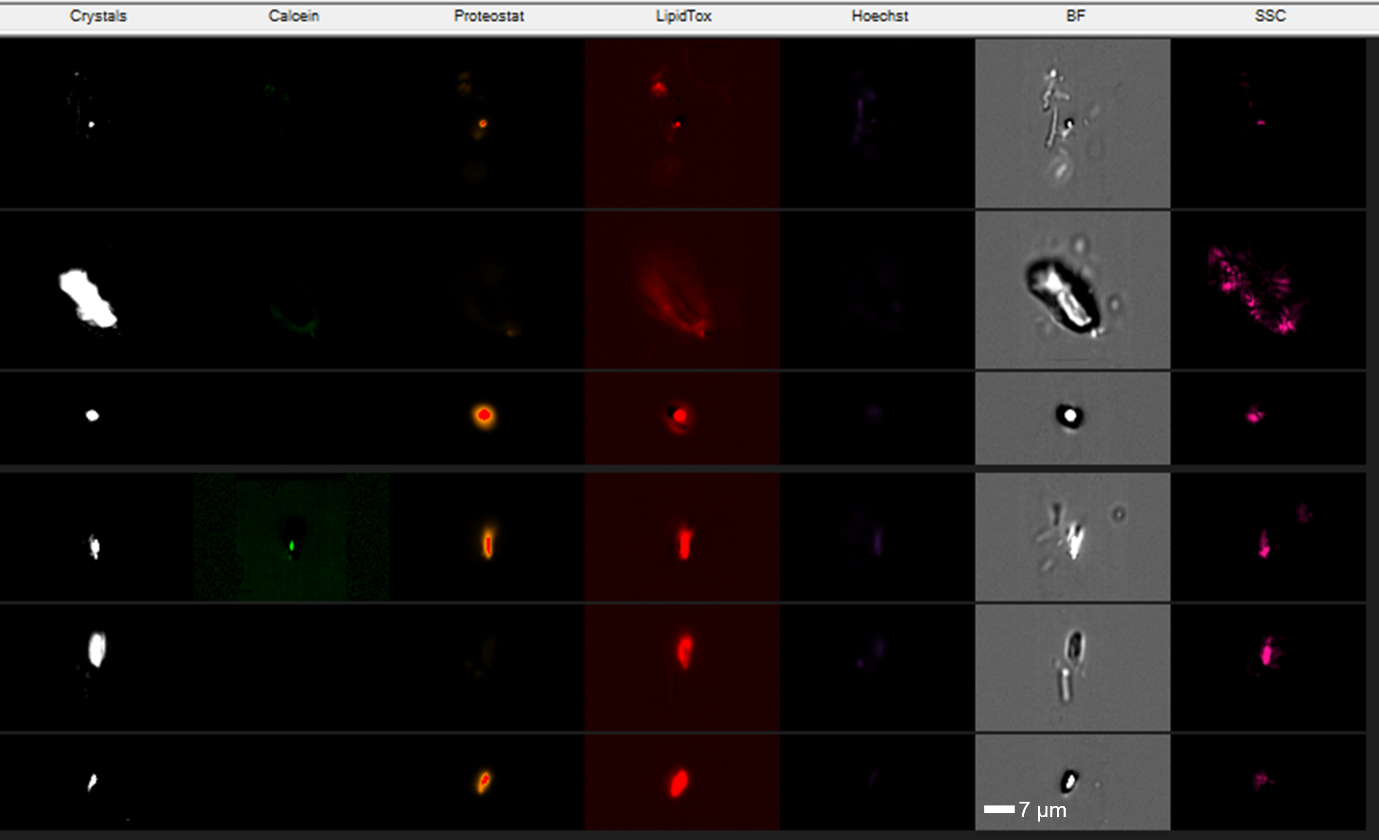


Figure S9. Particles found in O1 sample under the Birefringence by Area population definition. Each row represents one object, with information about the birefringence gate signal (crystals), Calcein, Proteostat, LipidTox and Hoechst signals as well as bright field (BF) and side scatter (SSC).

###

### Method 2: Birefringence by Calcein

The "Birefringence by Calcein" method relied on comparing the max pixel in the Calcein channel to that of the birefringence detection channel, with higher values considered part of the population. Using this approach, participant O1 again showed a notable concentration of approximately 1175 particles/mL, whereas other participants exhibited much lower concentrations, consistently below 150 particles/mL. The scatter graphs in Figure S10 indicate that the particles classified as crystals in the O1 samples were mostly located near the lower limit of the intensity range, suggesting that a slight adjustment in population boundaries could eliminate the apparent concentration spikes.


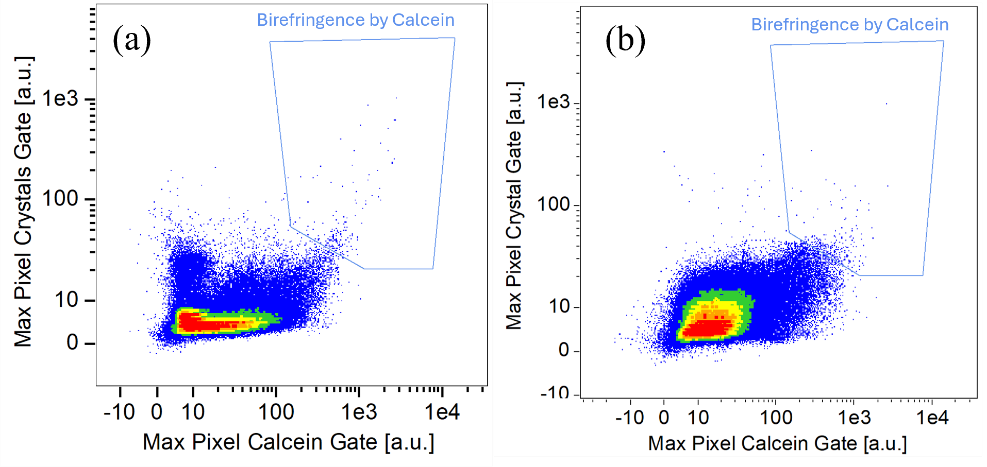


Figure S10. Population density scatter graphs of the measured object in IFC, max pixel Calcein vs. max pixel of particles in the birefringence gate of (a) typical sample and (b) F1 sample, taken from the “non-cells” population. Birefringence by Calcein population is marked on the figures.

The particles detected in the O1 samples under this definition are displayed in Figure S11. Similar to the "Birefringence by Area" classification, these particles showed contamination with LipidTox and Proteostat, as well as some bacterial signals detected by Hoechst staining. Although the Calcein signal was prominent, confirming the identity of these particles as true crystals remained challenging.


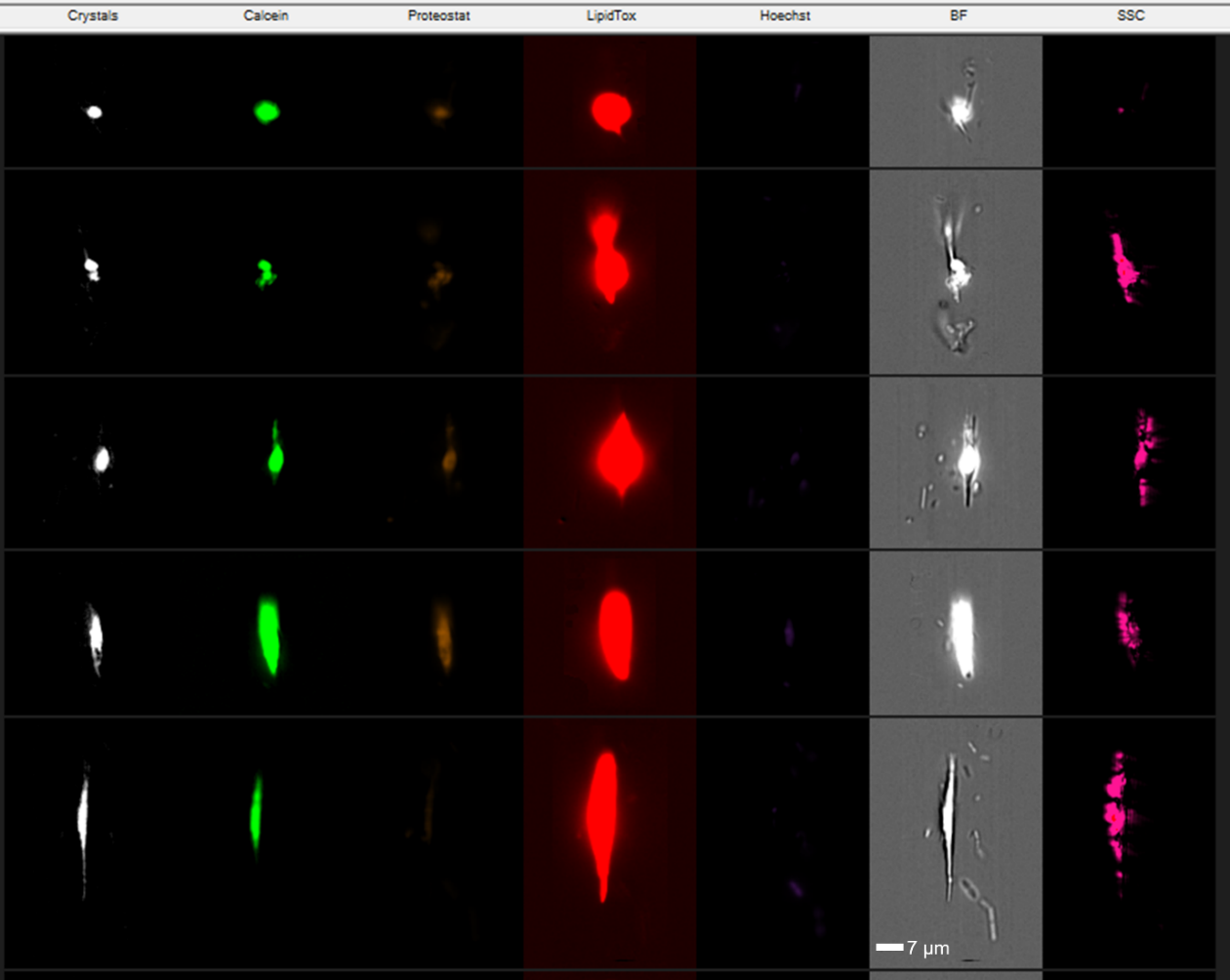


Figure S11. Particles found in O1 sample under the Birefringence by Calcein population definition. Each row represents one object, with information about the birefringence gate signal (crystals), Calcein, Proteostat, LipidTox and Hoechst signals as well as bright field (BF) and side scatter (SSC).

### Method 3: Only Birefringence

The "Only Birefringence" method focused on detecting particles with high birefringence channel intensity and minimal signal from other channels (LipidTox, Proteostat, and Hoechst), assuming that true crystals would not be tagged by non-Calcein markers. Under this definition, participants F9 and M7 displayed notable concentrations. Figure S12 shows scatter plots of the birefringence gate, illustrating distinct differences between typical samples, those from F9, and those from M7.


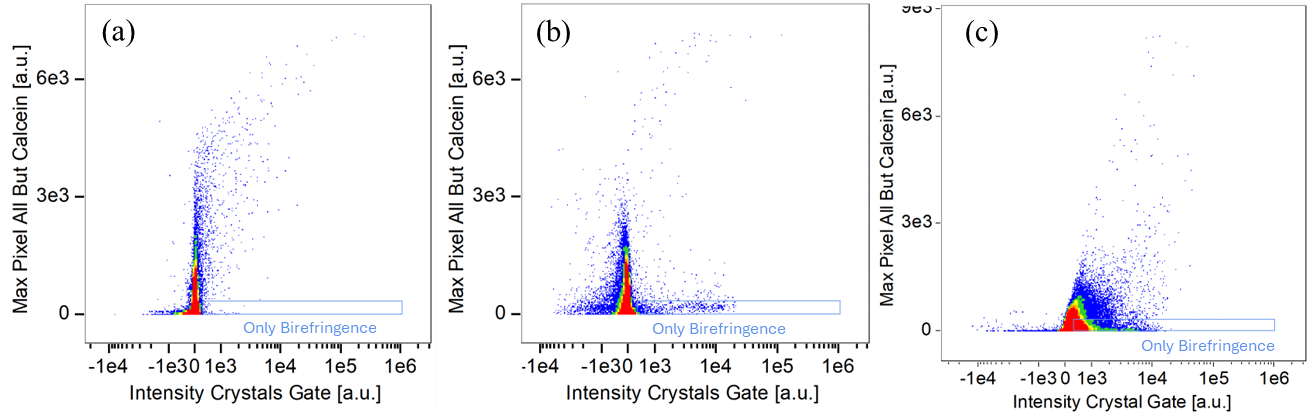


Figure S12. Population density scatter graphs of the measured object in IFC, intensity of particles in the birefringence gate vs. max pixel of LipidTox, Proteostat and Hoechst of (a) typical sample (b) F9 sample and (c) M7 sample, taken from the “non-cells” population. Only Birefringence population is marked on the figures.

Despite both participants meeting the "Only Birefringence" criteria, the nature of the detected particles varied significantly. Figure 13 compares the particles identified in samples from M7 and F9. The particles from F9 clearly exhibited features consistent with calcium oxalate (CaOx) crystals. In contrast, the particles found in sample from M7 could not be definitively identified as crystals.


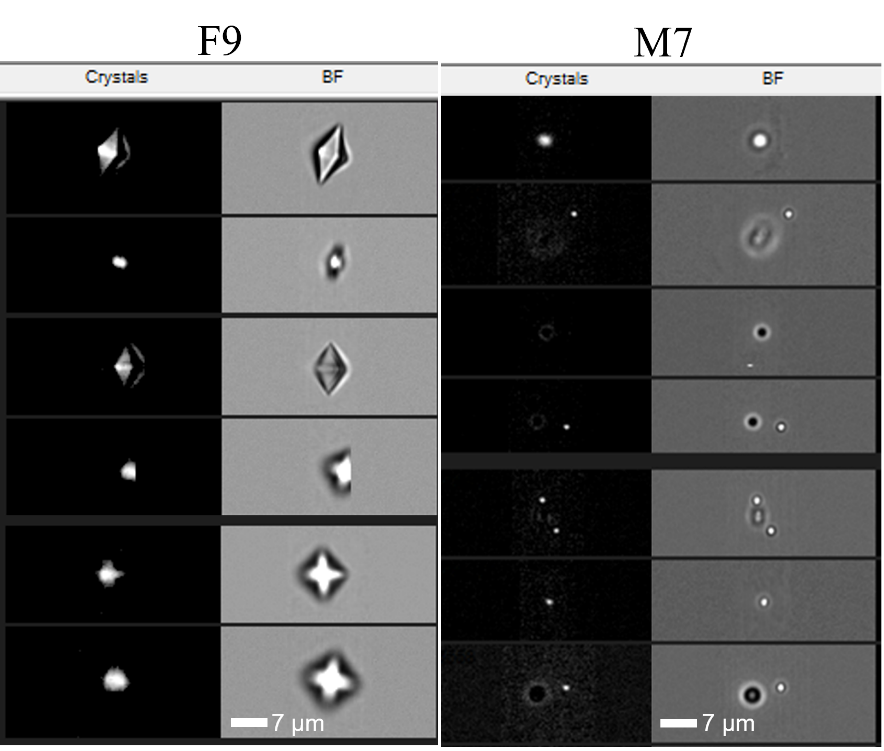


Figure S13. Particles found in F9 sample (left) and M7 sample (right) under the Only Birefringence population, shown in bright field (BF) and birefringence gate (crystals).

While the population definition of Only Birefringence discovered unidentified particles in M7, it fitted the CaOx in F9 samples very well. Scatter graphs of F9 samples of the other crystal definitions showed no difference from other typical samples, despite having proven crystals in the urine. Therefore, combined with the particles found in F1 samples above, the Only Birefringence definition is the best fit to estimate the crystals in the urine. However, manual validation remains essential to confirm the presence of crystals accurately.

## *Sample Collection – Storage and Preparation Time Effects, and Variability Among Samples from Same Donor*

The preparation time and collection tube types were initially tested on a single individual to develop a unified testing protocol. First-in-morning (FIM) samples were collected in vacuum tubes and Falcon vials and prepared three hours later (FIM) and seven hours later (late preparation, LP). The results are presented in Figure S14. Concentrations of the total particles and fluorescent-tagged particles used in the protocol development, grouped by sample time are shown in Section S.2, Table S4, while their mean areas are shown in Section S.2, Table S5.


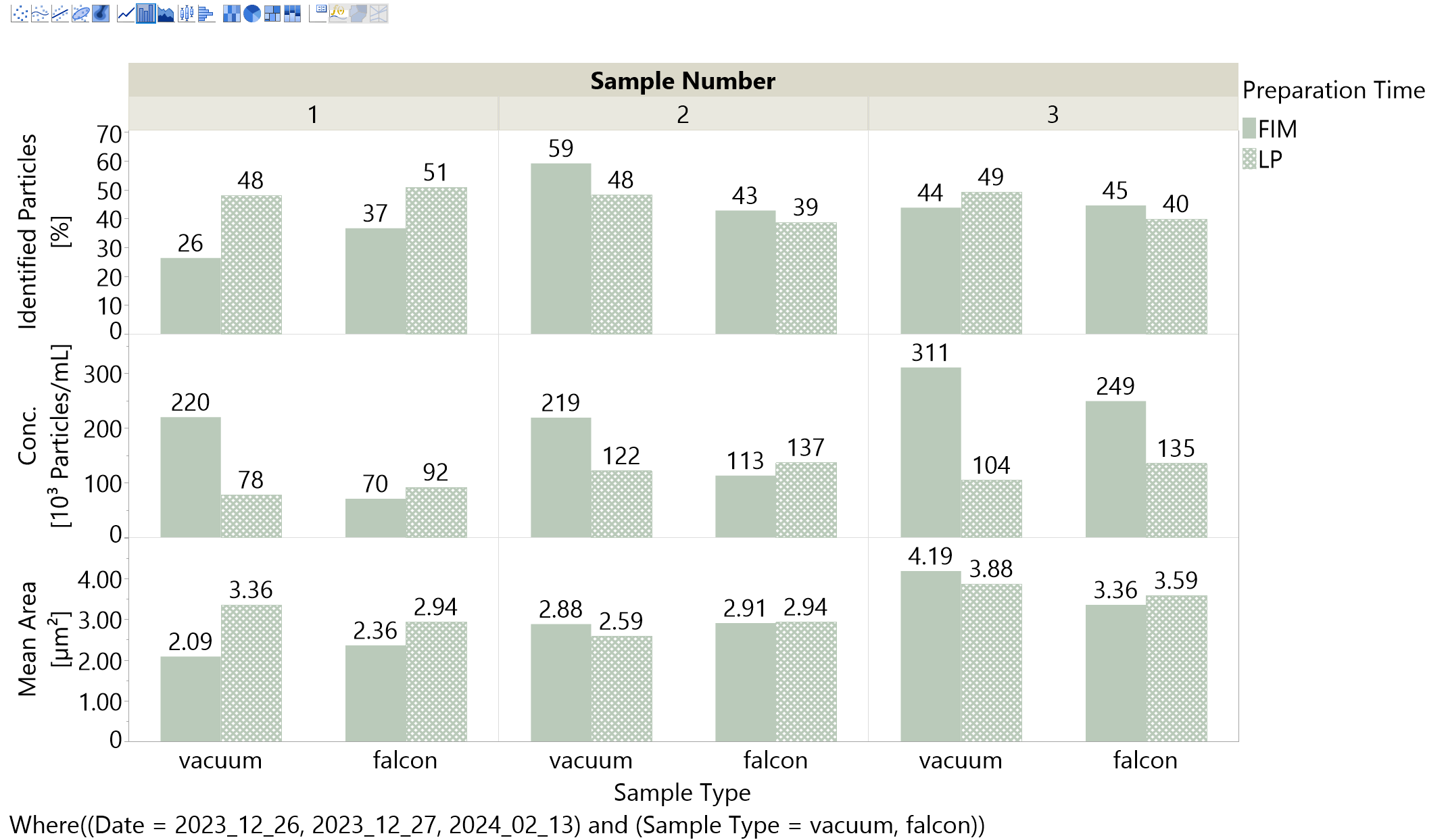


Figure S14. Comparative analysis of particulate matter characteristics in three urine samples from the same individual based on preparation time: 3 hours (FIM, solid) vs. 7 hours (LP, textured) post-collection and sample type (vacuum, left vs. Falcon tube, right) across three replications from samples collected on different days. The three rows represent different measured parameters. The top row (Identified Particles) represents the percentage of identifiable particles. The middle row (Conc.) illustrates the total number of particles detected per milliliter in the samples. The bottom row (Mean Area) depicts the mean area of all detected particles.

The comparison in Figure S14 showed natural variability amongst each set of three samples from each volunteer. For each parameter, different trends were noticed. In the first row, Identified Particles, generally, the LP samples showed a higher percentage than the morning samples, with variation across different sample types and replications.

For the concentration, a significant difference was observed between FIM and LP samples, particularly in the vacuum samples, where the morning preparation showed substantially higher concentrations. Falcon tube samples displayed less variability between the two preparation times but consistently lower concentrations than the vacuum samples. Following the drop in concentration, a standard preparation time of three hours was implemented for future samples.

Finally, for the average area, the LP samples generally had higher mean areas than the FIM samples, with some exceptions depending on the sample type and replication. Vacuum tubes consistently showed larger daily mean areas than Falcon tubes. The average increase in area could indicate aggregation over time, corresponding to the decrease in concentration. The graph shows the natural daily variability, which can be assumed to be a function of a combination of multiple factors, including diet, metabolism, and physical activity.

**NOTES FOR TABLES S4-14 and S16 BELOW:**

For individual results (Tables S4-S7, S16), the results show an average of 3 triplicates and standard deviation (STD). For population results (Tables S8-S14), where the individuals were grouped, standard error (SE) was used instead of STD. This was due to the results being a population means of triplicates of individual donors. SE = STD/√*n*, where $n$ is the number of samples.

## S.2 Sample Storage, Preparation Times, and Result Variability

Concentrations of the total particles and fluorescence tags used in the protocol development, grouped by preparation time, are shown in Table S4, while their mean areas are shown in Table S5. The results indicate the degree of variability among three samples from the same donor, collected on separate days.

Table S4. Mean ± STD and range (minimum, maximum values in parentheses) particle concentration results for different collection vessels at different preparation times for 3 samples of a single donor. FIM: first-in-morning sample with preparation 3 hours post collection, LP: late-preparation 7 hours post collection, FIM+LP: daily average.

| Vessel type | Preparation time | Identified Particles  (%) | Total particles  (10^3^ per mL) | Lipids  (10^3^ per mL) | Proteins  (10^3^ per mL) | Lipid-Proteins  (10^3^ per mL) | Calcium  (10^3^ per mL) | DNA  (per mL) |
| --- | --- | --- | --- | --- | --- | --- | --- | --- |
| Vacuum | FIM | 43 ± 13  (26, 59) | 250 ± 43  (219, 311) | 11 ± 1  (9, 13) | 58 ± 23  (26, 82) | 23 ± 21  (6, 53) | 16 ± 8  (5, 23) | 76 ± 41  (22, 121) |
|  | LP | 49 ± 1  (48, 49) | 101 ± 18  (78, 122) | 6 ± 1  (5, 8) | 22 ± 6  (17, 30) | 13 ± 9  (6, 25) | 9 ± 5  (4, 16) | 35 ± 19  (19, 62) |
|  | FIM+LP combined | 46 ± 10  (26, 59) | 176 ± 81  (78, 311) | 8 ± 3  (5, 13) | 40 ± 24  (17, 82) | 18 ± 17  (6, 53) | 13 ± 8  (4, 23) | 55 ± 38  (19, 121) |
| Falcon | FIM | 41 ± 3  (37, 45) | 144 ± 76  (70, 249) | 9 ± 2  (6, 10) | 27 ± 14  (12, 46) | 19 ± 21  (2, 49) | 8 ± 5  (5, 16) | 63 ± 50  (18, 133) |
|  | LP | 43 ± 5  (39, 51) | 121 ± 21  (92, 137) | 7 ± 2  (5, 9) | 22 ± 9  (12, 33) | 15 ± 8  (8, 26) | 9 ± 5  (5, 16) | 42 ± 10  (32, 56) |
|  | FIM+LP combined | 42 ± 5  (37, 51) | 133 ± 57  (70, 249) | 8 ± 2  (5, 10) | 24 ± 12  (12, 46) | 17 ± 16  (2, 49) | 9 ± 5  (5, 16) | 53 ± 38  (18, 133) |

Table S5. Mean ± STD and range (minimum, maximum values in parentheses) particle mean area results for different collection vessels at different preparation times for single donors. FIM: first-in-morning sample with preparation 3 hours post collection, LP: late-preparation 7 hours post collection, FIM+LP: daily average.

| Vessel type | Preparation time | Total particles  (µm^2^) | Lipids  (µm^2^) | Proteins  (µm^2^) | Lipid-Proteins  (µm^2^) | Calcium (µm^2^) | DNA  (µm^2^) |
| --- | --- | --- | --- | --- | --- | --- | --- |
| Vacuum | FIM | 3 ± 1  (2, 4) | 6.0 ± 0.4  (5.5, 6.5) | 8 ± 3  (5, 11) | 7 ± 1  (6, 9) | 10 ± 3  (6, 13) | 121 ± 16  (103, 141) |
|  | LP | 3 ± 1  (3, 4) | 5.7 ± 0.1  (5.6, 5.8) | 7 ± 2  (5, 9) | 6.3 ± 0.2  (6.1, 6.5) | 9 ± 2  (6, 11) | 130 ± 17  (107, 143) |
|  | FIM+LP combined | 3 ± 1  (2, 4) | 5.8 ± 0.3  (5.5, 6.5) | 7 ± 2  (5, 11) | 7 ± 1  (6, 9) | 10 ± 3  (6, 13) | 126 ± 17  (103, 143) |
| Falcon | FIM | 2.9 ± 0.4  (2.4, 3.4) | 5.5 ± 0.2  (5.4, 5.7) | 7 ± 2  (6, 10) | 7.8 ± 0.4  (7.4, 8.3) | 11 ± 4  (7, 17) | 117 ± 13  (103, 134) |
|  | LP | 3.2 ± 0.3  (2.9, 3.6) | 5.7 ± 0.2  (5.5, 5.9) | 7 ± 2  (5, 9) | 6 ± 1  (5, 7) | 11 ± 2  (9, 14) | 118 ± 16  (103, 139) |
|  | FIM+LP combined | 3.0 ± 0.4  (2.4, 3.6) | 5.6 ± 0.2  (5.4, 5.9) | 7 ± 2  (5, 10) | 7 ± 1  (5, 8) | 11 ± 3  (7, 17) | 117 ± 14  (103, 139) |

## S.3 Overall Results – Total Particles

Concentrations of the total particles and fluorescence tags used in the experiment for each participant separately, grouped by sample time, are shown in Table S6, while their mean areas are shown in Table S7.

Table S6. Mean ± STD and range (minimum, maximum values in parentheses) results of particle concentration for different times for 18 donors individually. FIM: first-in-morning sample, LM: late-morning samples, FIM+LM: daily average.

| Donor | Time of sample | Identified particles  (%) | Total particles  (10^3^ per mL) | Lipids  (10^3^ per mL) | Proteins  (10^3^ per mL) | Lipid-Proteins  (10^3^ per mL) | Calcium  (10^3^ per mL) | DNA  (per mL) |
| --- | --- | --- | --- | --- | --- | --- | --- | --- |
| Female 1 | FIM | 50.18 ± 6.87 (43.92, 57.53) | 792.73 ± 301.25 (506.39, 1106.96) | 8.46 ± 6.27 (3.02, 15.31) | 154.16 ± 69.50 (76.68, 211.02) | 159.63 ± 63.02 (92.52, 217.55) | 10.13 ± 4.14 (7.60, 14.90) | 20359.66 ± 4577.72 (16774.97, 25516.23) |
|  | LM | 55.34 ± 0.51 (54.80, 55.81) | 363.50 ± 82.77 (268.01, 414.86) | 10.60 ± 7.12 (5.58, 18.75) | 47.35 ± 29.09 (30.05, 80.93) | 133.27 ± 33.34 (98.46, 164.91) | 2.55 ± 0.64 (2.14, 3.28) | 10234.32 ± 5678.14 (5369.93, 16473.68) |
| Female 2 | FIM | 62.63 ± 2.32 (60.30, 64.94) | 154.74 ± 150.70 (48.97, 327.30) | 5.68 ± 7.65 (1.02, 14.50) | 32.51 ± 28.49 (9.04, 64.21) | 54.19 ± 50.18 (20.26, 111.83) | 1.31 ± 1.27 (0.44, 2.77) | 938.59 ± 1587.39 (12.69, 2771.51) |
|  | LM | 60.84 ± 12.12 (48.64, 72.87) | 90.03 ± 29.48 (57.97, 115.98) | 3.34 ± 1.64 (1.69, 4.97) | 26.40 ± 20.60 (7.35, 48.27) | 27.23 ± 7.73 (18.35, 32.50) | 0.61 ± 0.26 (0.31, 0.79) | 38.27 ± 16.39 (19.57, 50.13) |
| Female 3 | FIM | 61.14 ± 7.37 (56.49, 69.63) | 227.76 ± 31.75 (191.43, 250.19) | 13.60 ± 8.18 (4.44, 20.19) | 24.91 ± 5.04 (20.32, 30.30) | 97.46 ± 5.80 (91.39, 102.95) | 1.44 ± 0.43 (0.94, 1.74) | 127.78 ± 51.05 (75.61, 177.63) |
|  | LM | 57.82 ± 7.03 (51.14, 65.16) | 128.23 ± 115.41 (49.29, 260.68) | 8.98 ± 10.14 (2.92, 20.68) | 11.03 ± 7.39 (6.54, 19.56) | 58.15 ± 61.58 (17.25, 128.97) | 0.43 ± 0.01 (0.42, 0.44) | 53.55 ± 41.44 (17.98, 99.06) |
| Female 4 | FIM | 40.93 ± 4.13 (36.47, 44.62) | 175.43 ± 36.70 (134.57, 205.60) | 5.01 ± 0.25 (4.74, 5.24) | 18.32 ± 5.92 (12.53, 24.36) | 46.03 ± 2.79 (43.07, 48.62) | 1.63 ± 0.30 (1.30, 1.90) | 157.03 ± 136.34 (41.88, 307.58) |
|  | LM | 63.32 ± 7.83 (57.25, 72.16) | 159.22 ± 53.47 (116.53, 219.20) | 6.19 ± 5.05 (2.91, 12.01) | 17.23 ± 2.25 (15.06, 19.56) | 78.65 ± 44.09 (42.35, 127.72) | 1.21 ± 0.48 (0.87, 1.76) | 74.76 ± 23.24 (47.95, 89.04) |
| Female 5 | FIM | 49.86 ± 7.30 (41.59, 55.41) | 191.17 ± 84.24 (101.66, 268.90) | 7.49 ± 2.38 (5.83, 10.22) | 33.19 ± 11.88 (19.69, 42.04) | 35.02 ± 19.58 (21.25, 57.43) | 5.39 ± 6.84 (0.71, 13.24) | 314.82 ± 436.70 (44.22, 818.62) |
|  | LM | 52.48 ± 15.90 (35.38, 66.84) | 194.44 ± 61.50 (130.53, 253.21) | 11.05 ± 6.91 (3.47, 16.98) | 48.86 ± 45.11 (19.92, 100.84) | 41.56 ± 13.05 (28.55, 54.65) | 0.56 ± 0.45 (0.19, 1.06) | 108.14 ± 25.01 (86.09, 135.31) |
| Female 6 | FIM | 39.52 ± 12.61 (25.35, 49.52) | 229.71 ± 52.88 (192.74, 290.28) | 6.61 ± 1.85 (4.84, 8.54) | 20.26 ± 5.51 (16.54, 26.59) | 55.33 ± 12.31 (43.53, 68.09) | 2.76 ± 2.61 (0.96, 5.76) | 355.32 ± 174.65 (174.06, 522.51) |
|  | LM | 48.89 ± 11.62 (39.29, 61.81) | 228.94 ± 150.65 (91.94, 390.27) | 11.61 ± 2.05 (9.33, 13.29) | 20.35 ± 19.87 (4.05, 42.48) | 89.02 ± 81.72 (22.08, 180.09) | 0.95 ± 0.30 (0.60, 1.16) | 201.50 ± 173.91 (24.45, 372.09) |
| Female 7 | FIM | 58.85 ± 9.14 (52.94, 69.37) | 151.28 ± 35.40 (122.40, 190.78) | 8.57 ± 1.16 (7.23, 9.26) | 25.68 ± 7.55 (17.45, 32.29) | 53.07 ± 15.29 (41.19, 70.32) | 0.97 ± 0.70 (0.41, 1.75) | 203.14 ± 133.30 (117.28, 356.70) |
|  | LM | 60.03 ± 7.91 (51.49, 67.12) | 200.50 ± 135.40 (64.93, 335.72) | 11.59 ± 7.24 (3.97, 18.37) | 38.10 ± 38.13 (5.72, 80.12) | 74.48 ± 49.43 (23.06, 121.65) | 1.30 ± 0.64 (0.63, 1.91) | 1312.21 ± 1301.66 (9.05, 2612.36) |
| Female 8 | FIM | 54.79 ± 9.51 (45.20, 64.23) | 90.92 ± 38.65 (46.93, 119.43) | 3.03 ± 1.95 (1.27, 5.12) | 15.73 ± 11.14 (5.14, 27.34) | 31.60 ± 15.92 (13.33, 42.47) | 1.19 ± 0.18 (1.05, 1.39) | 172.37 ± 160.82 (27.30, 345.30) |
|  | LM | 52.57 ± 0.79 (52.06, 53.48) | 140.46 ± 14.22 (124.70, 152.34) | 4.76 ± 0.92 (4.03, 5.80) | 19.70 ± 5.79 (13.89, 25.46) | 44.86 ± 2.14 (42.48, 46.61) | 3.49 ± 1.58 (1.82, 4.96) | 525.16 ± 93.81 (417.17, 586.51) |
| Female 9 | FIM | 45.42 ± 10.94 (37.44, 57.90) | 254.84 ± 78.87 (175.62, 333.35) | 3.58 ± 1.08 (2.66, 4.76) | 27.72 ± 9.84 (17.93, 37.61) | 62.73 ± 31.58 (33.30, 96.10) | 6.23 ± 1.82 (4.20, 7.71) | 14314.23 ± 19611.52 (1486.03, 36889.68) |
|  | LM | 49.80 ± 8.70 (42.57, 59.46) | 364.31 ± 197.51 (145.56, 529.55) | 14.32 ± 8.49 (5.45, 22.37) | 36.08 ± 21.49 (13.58, 56.40) | 110.48 ± 47.37 (64.34, 159.00) | 6.51 ± 3.65 (2.75, 10.03) | 1417.78 ± 1421.80 (387.62, 3039.93) |
| Male 1 | FIM | 55.94 ± 8.17 (49.15, 65.02) | 111.32 ± 21.52 (93.38, 135.18) | 5.75 ± 2.94 (3.81, 9.13) | 19.59 ± 13.70 (9.26, 35.13) | 36.62 ± 10.48 (26.30, 47.26) | 1.48 ± 0.47 (1.11, 2.01) | 44.12 ± 14.76 (28.99, 58.47) |
|  | LM | 56.79 ± 5.43 (50.84, 61.49) | 232.39 ± 126.32 (92.50, 338.12) | 25.55 ± 16.96 (6.06, 36.97) | 26.59 ± 13.68 (12.06, 39.21) | 82.42 ± 47.76 (28.46, 119.23) | 2.03 ± 1.38 (0.48, 3.11) | 43.33 ± 15.28 (26.52, 56.37) |
| Male 2 | FIM | 37.62 ± 18.73 (21.44, 58.14) | 226.77 ± 121.97 (92.20, 330.04) | 10.35 ± 5.50 (4.45, 15.34) | 22.97 ± 10.14 (13.11, 33.36) | 37.38 ± 20.02 (19.03, 58.73) | 2.25 ± 0.85 (1.40, 3.10) | 148.35 ± 113.54 (28.04, 253.63) |
|  | LM | 54.98 ± 4.84 (49.50, 58.69) | 95.96 ± 32.69 (70.63, 132.86) | 7.10 ± 6.08 (3.46, 14.12) | 11.36 ± 2.20 (8.82, 12.71) | 32.95 ± 10.91 (24.81, 45.34) | 1.27 ± 1.34 (0.48, 2.81) | 37.38 ± 35.53 (15.15, 78.36) |
| Male 3 | FIM | 35.33 ± 3.33 (32.77, 39.10) | 241.99 ± 110.14 (173.20, 369.03) | 21.17 ± 2.96 (18.20, 24.12) | 25.93 ± 22.86 (11.66, 52.29) | 32.99 ± 10.64 (24.70, 44.98) | 3.87 ± 2.46 (2.00, 6.66) | 348.25 ± 258.33 (85.83, 602.29) |
|  | LM | 59.70 ± 1.59 (58.70, 61.53) | 308.95 ± 100.27 (227.65, 420.99) | 96.63 ± 31.72 (69.22, 131.37) | 18.66 ± 5.80 (12.10, 23.08) | 65.41 ± 27.54 (35.45, 89.63) | 3.13 ± 0.94 (2.33, 4.16) | 167.49 ± 81.66 (81.06, 243.35) |
| Male 4 | FIM | 32.26 ± 10.45 (24.70, 44.19) | 220.68 ± 39.97 (174.73, 247.42) | 10.46 ± 3.05 (7.03, 12.85) | 25.84 ± 11.52 (12.54, 32.88) | 21.28 ± 0.67 (20.67, 22.00) | 5.78 ± 3.07 (2.87, 8.99) | 95.15 ± 43.67 (44.90, 123.96) |
|  | LM | 36.94 ± 11.56 (25.12, 48.21) | 435.33 ± 348.13 (105.37, 799.16) | 24.37 ± 14.04 (15.51, 40.56) | 68.95 ± 61.13 (16.44, 136.05) | 53.16 ± 54.94 (15.65, 116.22) | 3.81 ± 2.19 (1.68, 6.06) | 159.22 ± 189.70 (10.30, 372.79) |
| Male 5 | FIM | 27.07 ± 4.72 (21.85, 31.02) | 211.73 ± 103.82 (91.85, 272.13) | 17.00 ± 8.35 (11.33, 26.59) | 17.10 ± 17.18 (2.23, 35.91) | 15.56 ± 8.00 (9.77, 24.69) | 10.37 ± 10.68 (3.37, 22.66) | 70.36 ± 36.42 (31.69, 104.01) |
|  | LM | 42.11 ± 4.83 (36.83, 46.30) | 342.77 ± 173.35 (195.74, 533.91) | 43.10 ± 22.19 (18.71, 62.10) | 40.49 ± 37.65 (15.48, 83.79) | 51.21 ± 18.59 (32.70, 69.88) | 11.01 ± 6.21 (4.12, 16.18) | 244.68 ± 203.27 (20.55, 417.12) |
| Male 6 | FIM | 18.96 ± 5.60 (14.37, 25.19) | 175.41 ± 59.09 (117.83, 235.91) | 3.81 ± 0.30 (3.60, 4.15) | 9.17 ± 6.16 (4.48, 16.14) | 13.83 ± 9.52 (5.85, 24.37) | 4.07 ± 1.61 (2.83, 5.89) | 65.06 ± 42.64 (32.35, 113.29) |
|  | LM | 38.38 ± 6.80 (31.55, 45.15) | 142.37 ± 59.00 (74.79, 183.60) | 11.99 ± 5.44 (8.57, 18.26) | 17.46 ± 7.98 (8.84, 24.60) | 18.48 ± 6.68 (14.44, 26.19) | 3.83 ± 2.24 (1.26, 5.33) | 81.24 ± 32.01 (60.59, 118.11) |
| Male 7 | FIM | 28.90 ± 6.28 (22.64, 35.21) | 224.33 ± 80.09 (131.86, 271.75) | 12.06 ± 3.28 (8.46, 14.88) | 24.24 ± 18.13 (6.49, 42.73) | 18.47 ± 9.24 (11.25, 28.89) | 9.97 ± 3.81 (7.39, 14.34) | 269.60 ± 321.54 (60.76, 639.87) |
|  | LM | 31.40 ± 7.84 (26.14, 40.41) | 185.51 ± 24.05 (157.93, 202.08) | 15.75 ± 6.24 (11.24, 22.87) | 10.55 ± 2.76 (7.37, 12.30) | 26.10 ± 8.50 (16.56, 32.84) | 4.62 ± 3.81 (1.82, 8.95) | 72.77 ± 38.81 (46.48, 117.34) |
| Male 8 | FIM | 25.19 ± 7.63 (17.87, 33.10) | 257.54 ± 26.42 (235.91, 286.99) | 7.16 ± 3.23 (3.43, 9.08) | 31.16 ± 11.06 (19.00, 40.61) | 23.97 ± 7.20 (17.03, 31.41) | 2.06 ± 0.62 (1.40, 2.62) | 59.07 ± 9.70 (48.03, 66.22) |
|  | LM | 34.38 ± 22.38 (17.62, 59.79) | 232.19 ± 119.09 (137.72, 365.96) | 9.63 ± 5.70 (6.33, 16.22) | 32.11 ± 17.53 (11.88, 42.71) | 25.27 ± 10.92 (12.66, 31.93) | 2.99 ± 0.94 (2.06, 3.93) | 41.07 ± 4.96 (35.68, 45.45) |
| Male 9 | FIM | 54.12 ± 9.44 (44.17, 62.95) | 171.33 ± 97.48 (91.02, 279.79) | 10.47 ± 6.52 (3.57, 16.54) | 30.54 ± 22.06 (12.90, 55.27) | 39.38 ± 12.19 (26.71, 51.03) | 4.95 ± 2.50 (2.57, 7.56) | 88.33 ± 33.70 (67.07, 127.18) |
|  | LM | 44.49 ± 19.38 (22.91, 60.41) | 179.79 ± 84.11 (113.53, 274.42) | 15.58 ± 14.06 (1.64, 29.75) | 20.39 ± 14.02 (8.21, 35.71) | 39.73 ± 22.82 (14.25, 58.29) | 9.90 ± 7.66 (1.09, 15.01) | 69.36 ± 78.03 (9.78, 157.69) |
| Outlier 1 | FIM | 53.31 ± 7.50 (44.67, 58.15) | 2227.54 ± 2307.37 (478.74, 4842.69) | 53.55 ± 55.25 (17.31, 117.14) | 306.85 ± 266.20 (56.01, 586.13) | 447.82 ± 427.05 (185.11, 940.57) | 27.68 ± 24.24 (2.45, 50.80) | 40719.61 ± 39541.59 (2230.95, 81236.10) |
|  | LM | 50.71 ± 13.44 (39.98, 65.78) | 605.45 ± 635.71 (233.06, 1339.47) | 23.94 ± 11.09 (15.56, 36.52) | 127.88 ± 137.75 (23.99, 284.13) | 224.31 ± 268.23 (56.15, 533.64) | 2.39 ± 1.66 (1.13, 4.27) | 5089.45 ± 7954.45 (146.83, 14265.32) |

Table S7. Mean ± STD and range (minimum, maximum values in parentheses) results of particle mean area for different times for 18 donors individually. FIM: first-in-morning sample, LM: late-morning samples, FIM+LM: daily average.

| Donor | Time of sample | Total particles  (µm^2^) | Lipids  (µm^2^) | Proteins  (µm^2^) | Lipid-Proteins  (µm^2^) | Calcium  (µm^2^) | DNA  (µm^2^) |
| --- | --- | --- | --- | --- | --- | --- | --- |
| Female 1 | FIM | 6.22 ± 0.17 (6.08, 6.42) | 6.39 ± 0.23 (6.22, 6.65) | 12.30 ± 2.13 (11.02, 14.76) | 20.97 ± 1.12 (19.92, 22.15) | 8.75 ± 2.96 (6.53, 12.11) | 115.54 ± 3.33 (111.70, 117.59) |
|  | LM | 8.91 ± 0.98 (7.86, 9.80) | 6.23 ± 0.32 (5.93, 6.56) | 13.33 ± 0.95 (12.43, 14.32) | 21.86 ± 2.36 (19.51, 24.22) | 6.88 ± 1.21 (5.99, 8.26) | 67.95 ± 3.71 (64.88, 72.07) |
| Female 2 | FIM | 4.38 ± 0.45 (3.99, 4.87) | 7.64 ± 2.51 (6.03, 10.54) | 11.29 ± 1.53 (9.57, 12.49) | 8.84 ± 1.39 (8.00, 10.45) | 6.34 ± 1.10 (5.22, 7.43) | 140.23 ± 44.51 (89.25, 171.38) |
|  | LM | 4.58 ± 0.54 (4.25, 5.20) | 7.62 ± 1.46 (6.39, 9.24) | 12.20 ± 3.62 (9.29, 16.26) | 10.39 ± 0.99 (9.53, 11.47) | 7.38 ± 0.36 (6.96, 7.60) | 266.66 ± 80.59 (174.27, 322.52) |
| Female 3 | FIM | 4.96 ± 0.33 (4.71, 5.33) | 9.47 ± 2.02 (7.66, 11.65) | 13.66 ± 2.53 (10.77, 15.47) | 11.30 ± 2.58 (8.46, 13.50) | 13.35 ± 1.77 (11.57, 15.10) | 360.36 ± 269.52 (163.87, 667.62) |
|  | LM | 4.70 ± 0.51 (4.16, 5.17) | 7.62 ± 1.15 (6.55, 8.84) | 7.18 ± 2.99 (3.76, 9.29) | 11.10 ± 1.99 (9.64, 13.37) | 16.34 ± 5.19 (10.57, 20.64) | 156.16 ± 24.71 (128.69, 176.59) |
| Female 4 | FIM | 4.30 ± 1.17 (3.00, 5.28) | 7.40 ± 0.02 (7.38, 7.41) | 11.77 ± 2.30 (9.47, 14.07) | 10.76 ± 3.02 (8.61, 14.21) | 10.26 ± 0.61 (9.72, 10.91) | 319.75 ± 198.10 (201.32, 548.45) |
|  | LM | 6.07 ± 0.91 (5.03, 6.72) | 7.36 ± 0.99 (6.26, 8.18) | 11.92 ± 1.25 (11.04, 13.35) | 15.42 ± 4.72 (10.08, 19.05) | 8.68 ± 1.21 (7.33, 9.67) | 348.45 ± 111.67 (227.04, 446.77) |
| Female 5 | FIM | 5.34 ± 0.86 (4.35, 5.91) | 6.51 ± 1.08 (5.66, 7.72) | 12.70 ± 2.26 (10.18, 14.55) | 12.47 ± 2.29 (10.37, 14.90) | 6.92 ± 1.09 (5.96, 8.11) | 213.16 ± 107.29 (126.91, 333.31) |
|  | LM | 5.53 ± 1.42 (3.90, 6.42) | 6.47 ± 0.96 (5.40, 7.23) | 12.03 ± 1.48 (10.38, 13.26) | 12.74 ± 3.02 (9.27, 14.76) | 5.81 ± 0.31 (5.50, 6.12) | 237.16 ± 188.34 (88.38, 448.92) |
| Female 6 | FIM | 4.03 ± 1.49 (2.52, 5.50) | 6.09 ± 0.79 (5.39, 6.95) | 12.19 ± 1.02 (11.08, 13.07) | 10.15 ± 0.46 (9.77, 10.66) | 9.65 ± 1.45 (8.60, 11.31) | 430.29 ± 291.70 (99.50, 650.69) |
|  | LM | 5.24 ± 0.71 (4.58, 6.00) | 5.80 ± 0.39 (5.37, 6.12) | 7.56 ± 3.42 (4.03, 10.87) | 10.50 ± 2.05 (9.18, 12.87) | 8.31 ± 1.25 (7.17, 9.64) | 331.20 ± 137.14 (174.08, 426.89) |
| Female 7 | FIM | 4.86 ± 0.50 (4.41, 5.39) | 8.97 ± 2.73 (6.76, 12.02) | 12.90 ± 2.60 (10.03, 15.09) | 11.23 ± 0.94 (10.34, 12.21) | 19.53 ± 2.49 (17.45, 22.29) | 168.96 ± 42.37 (135.31, 216.55) |
|  | LM | 4.63 ± 0.45 (4.30, 5.14) | 9.49 ± 4.91 (6.12, 15.13) | 9.03 ± 0.40 (8.78, 9.49) | 10.34 ± 1.23 (9.55, 11.76) | 12.44 ± 6.06 (7.90, 19.32) | 169.94 ± 96.16 (100.08, 279.61) |
| Female 8 | FIM | 5.32 ± 1.01 (4.32, 6.33) | 7.51 ± 0.30 (7.17, 7.70) | 11.26 ± 2.92 (9.55, 14.63) | 9.54 ± 1.82 (7.44, 10.59) | 9.75 ± 0.48 (9.20, 10.06) | 327.44 ± 158.76 (198.30, 504.69) |
|  | LM | 4.71 ± 0.23 (4.55, 4.98) | 7.37 ± 1.33 (6.54, 8.90) | 8.79 ± 1.34 (7.99, 10.34) | 10.17 ± 1.27 (8.79, 11.28) | 7.60 ± 2.42 (5.18, 10.01) | 218.18 ± 74.21 (142.43, 290.75) |
| Female 9 | FIM | 8.76 ± 6.16 (4.01, 15.72) | 7.60 ± 2.06 (5.90, 9.88) | 10.23 ± 1.76 (8.46, 11.97) | 10.79 ± 1.80 (9.10, 12.68) | 9.35 ± 1.82 (7.49, 11.13) | 156.36 ± 26.03 (130.19, 182.25) |
|  | LM | 4.27 ± 0.47 (3.73, 4.55) | 7.75 ± 2.68 (6.16, 10.84) | 8.98 ± 2.17 (6.73, 11.06) | 9.82 ± 1.96 (7.83, 11.75) | 12.11 ± 5.11 (6.23, 15.55) | 282.62 ± 112.41 (215.38, 412.39) |
| Male 1 | FIM | 4.13 ± 0.62 (3.55, 4.79) | 7.24 ± 0.54 (6.88, 7.86) | 8.88 ± 3.75 (6.04, 13.14) | 8.17 ± 1.33 (7.25, 9.71) | 7.33 ± 0.24 (7.06, 7.51) | 183.77 ± 22.98 (160.81, 206.78) |
|  | LM | 4.17 ± 0.49 (3.73, 4.70) | 6.41 ± 0.54 (5.93, 6.99) | 9.10 ± 0.75 (8.23, 9.57) | 8.44 ± 0.86 (7.49, 9.16) | 7.23 ± 2.09 (5.39, 9.50) | 199.86 ± 3.69 (195.63, 202.48) |
| Male 2 | FIM | 3.25 ± 0.90 (2.59, 4.28) | 8.05 ± 1.33 (7.08, 9.57) | 13.30 ± 3.51 (9.31, 15.92) | 10.23 ± 1.71 (8.67, 12.06) | 9.38 ± 1.80 (7.38, 10.85) | 180.68 ± 25.22 (158.47, 208.09) |
|  | LM | 4.20 ± 0.24 (3.94, 4.42) | 7.41 ± 1.18 (6.17, 8.54) | 8.87 ± 0.78 (8.19, 9.72) | 9.38 ± 0.78 (8.90, 10.27) | 6.84 ± 1.29 (5.89, 8.31) | 232.47 ± 22.00 (209.43, 253.26) |
| Male 3 | FIM | 3.39 ± 0.86 (2.71, 4.35) | 7.56 ± 0.72 (6.75, 8.13) | 12.05 ± 3.70 (7.91, 15.03) | 9.79 ± 1.58 (8.25, 11.40) | 9.53 ± 0.32 (9.26, 9.88) | 187.72 ± 84.38 (90.31, 238.34) |
|  | LM | 3.84 ± 0.29 (3.65, 4.17) | 6.96 ± 0.15 (6.82, 7.12) | 9.26 ± 0.98 (8.31, 10.27) | 10.21 ± 0.44 (9.75, 10.62) | 6.98 ± 0.98 (6.15, 8.05) | 175.93 ± 29.73 (141.63, 194.35) |
| Male 4 | FIM | 2.77 ± 0.39 (2.49, 3.21) | 6.30 ± 0.67 (5.55, 6.84) | 8.80 ± 0.66 (8.04, 9.23) | 8.88 ± 0.72 (8.10, 9.52) | 8.13 ± 2.64 (6.58, 11.18) | 155.58 ± 48.16 (120.14, 210.41) |
|  | LM | 2.57 ± 0.66 (2.17, 3.33) | 5.65 ± 0.61 (4.96, 6.10) | 9.33 ± 2.17 (6.83, 10.63) | 10.15 ± 1.78 (8.47, 12.02) | 8.04 ± 2.22 (5.57, 9.87) | 171.31 ± 11.21 (163.73, 184.19) |
| Male 5 | FIM | 2.56 ± 0.48 (2.18, 3.10) | 5.69 ± 0.35 (5.30, 5.97) | 7.48 ± 0.94 (6.41, 8.17) | 8.17 ± 0.92 (7.46, 9.21) | 9.76 ± 3.96 (7.08, 14.30) | 263.96 ± 103.71 (156.95, 364.02) |
|  | LM | 3.42 ± 0.67 (2.67, 3.92) | 5.72 ± 0.17 (5.52, 5.84) | 10.29 ± 5.19 (7.22, 16.28) | 12.09 ± 3.95 (9.78, 16.65) | 8.43 ± 1.52 (7.16, 10.11) | 155.44 ± 27.09 (124.21, 172.72) |
| Male 6 | FIM | 1.85 ± 0.28 (1.58, 2.15) | 6.59 ± 0.14 (6.46, 6.74) | 7.46 ± 0.85 (6.51, 8.16) | 7.29 ± 0.44 (7.02, 7.79) | 7.00 ± 1.26 (5.58, 8.00) | 122.52 ± 9.77 (111.67, 130.62) |
|  | LM | 2.71 ± 0.82 (1.88, 3.51) | 5.75 ± 0.50 (5.45, 6.33) | 6.28 ± 1.51 (4.87, 7.88) | 8.45 ± 0.57 (7.82, 8.94) | 6.37 ± 0.02 (6.35, 6.38) | 154.34 ± 28.37 (132.07, 186.28) |
| Male 7 | FIM | 2.30 ± 0.20 (2.12, 2.51) | 5.66 ± 0.21 (5.42, 5.79) | 10.93 ± 2.59 (7.95, 12.66) | 8.81 ± 1.01 (8.04, 9.96) | 7.28 ± 0.65 (6.64, 7.95) | 121.82 ± 50.98 (74.21, 175.60) |
|  | LM | 2.35 ± 0.46 (1.84, 2.71) | 5.25 ± 0.29 (5.00, 5.56) | 6.27 ± 1.57 (5.14, 8.06) | 9.53 ± 2.60 (7.58, 12.47) | 7.32 ± 1.39 (6.38, 8.91) | 210.93 ± 19.38 (191.96, 230.69) |
| Male 8 | FIM | 2.20 ± 0.51 (1.65, 2.66) | 8.36 ± 1.68 (6.50, 9.75) | 13.76 ± 0.31 (13.43, 14.03) | 10.20 ± 1.63 (8.55, 11.82) | 11.37 ± 0.95 (10.36, 12.25) | 156.61 ± 22.33 (131.03, 172.16) |
|  | LM | 2.58 ± 1.53 (1.36, 4.30) | 7.64 ± 1.71 (5.73, 9.02) | 12.40 ± 2.39 (10.03, 14.81) | 9.92 ± 1.62 (8.59, 11.72) | 9.97 ± 2.98 (7.52, 13.29) | 185.96 ± 49.84 (137.72, 237.25) |
| Male 9 | FIM | 4.09 ± 0.28 (3.77, 4.30) | 6.22 ± 0.47 (5.95, 6.76) | 9.81 ± 1.61 (7.95, 10.79) | 7.83 ± 0.77 (7.00, 8.52) | 11.07 ± 3.60 (7.72, 14.88) | 115.53 ± 20.02 (94.26, 134.00) |
|  | LM | 4.28 ± 1.14 (3.15, 5.43) | 5.98 ± 1.42 (4.75, 7.53) | 9.49 ± 2.95 (6.74, 12.60) | 7.68 ± 0.68 (7.22, 8.45) | 11.78 ± 2.69 (8.71, 13.73) | 155.34 ± 45.36 (108.75, 199.35) |
| Outlier 1 | FIM | 6.90 ± 1.52 (5.64, 8.59) | 7.23 ± 0.38 (7.00, 7.67) | 12.74 ± 1.81 (10.71, 14.17) | 21.91 ± 7.63 (13.41, 28.18) | 9.80 ± 4.49 (5.82, 14.67) | 163.09 ± 25.40 (137.13, 187.89) |
|  | LM | 5.28 ± 1.55 (4.20, 7.06) | 6.77 ± 1.36 (5.93, 8.34) | 10.35 ± 1.34 (9.15, 11.80) | 14.73 ± 7.92 (9.89, 23.87) | 6.52 ± 1.26 (5.31, 7.82) | 106.83 ± 33.37 (81.93, 144.75) |

## S.4 Overall Results Grouped Data by Sample Time

Concentrations and mean areas of the total particles and fluorescence tags used in the experiment, averaged over 18 participants and grouped by sample time, are shown in Table S8.

Table S8. Mean ± SE and range (minimum, maximum values in parentheses) results for different times for 18 donors. FIM: first-in-morning sample, LM: late-morning samples, FIM+LM: daily average.

| Category | Collection time | Total particles | Lipids | Proteins | Lipid-Proteins | Calcium | DNA |
| --- | --- | --- | --- | --- | --- | --- | --- |
| Identified Particles (%) | FIM | 43.26 ± 5.69 (14.37, 69.63) |  |  |  |  |  |
|  | LM | 50.01 ± 4.90 (17.62, 72.87) |  |  |  |  |  |
|  | FIM+LM combined | 46.64 ± 5.44 (14.37, 72.87) |  |  |  |  |  |
| Concentration  (NOTE: 10^3^ per mL except for  DNA: per mL) | FIM | 228.32 ± 38.77 (46.93, 1106.96) | 8.90 ± 1.23 (1.02, 26.59) | 31.06 ± 6.97 (2.23, 211.02) | 46.36 ± 7.38 (5.85, 217.55) | 4.21 ± 0.90 (0.41, 22.66) | 2118.40 ± 1479.85 (12.69, 36889.68) |
|  | LM | 223.61 ± 33.61 (49.29, 799.16) | 18.45 ± 4.79 (1.64, 131.37) | 28.43 ± 5.11 (4.05, 136.05) | 58.47 ± 8.28 (12.66, 180.09) | 3.34 ± 0.79 (0.19, 16.18) | 826.79 ± 570.39 (9.05, 16473.68) |
|  | FIM+LM combined | 225.96 ± 36.12 (46.93, 1106.96) | 13.68 ± 3.62 (1.02, 131.37) | 29.74 ± 6.09 (2.23, 211.02) | 52.42 ± 7.89 (5.85, 217.55) | 3.78 ± 0.85 (0.19, 22.66) | 1472.60 ± 1125.14 (9.05, 36889.68) |
| Mean Area  (µm^2^) | FIM | 4.15 ± 0.55 (1.58, 15.72) | 7.18 ± 0.34 (5.30, 12.02) | 11.15 ± 0.58 (6.04, 15.92) | 10.30 ± 0.66 (7.00, 22.15) | 9.71 ± 0.78 (5.22, 22.29) | 206.68 ± 32.93 (74.21, 667.62) |
|  | LM | 4.38 ± 0.43 (1.36, 9.80) | 6.80 ± 0.38 (4.75, 15.13) | 9.57 ± 0.60 (3.76, 16.28) | 11.01 ± 0.74 (7.22, 24.22) | 8.81 ± 0.80 (5.18, 20.64) | 206.66 ± 22.60 (64.88, 448.92) |
|  | FIM+LM combined | 4.26 ± 0.49 (1.36, 15.72) | 6.99 ± 0.36 (4.75, 15.13) | 10.36 ± 0.61 (3.76, 16.28) | 10.66 ± 0.70 (7.00, 24.22) | 9.26 ± 0.79 (5.18, 22.29) | 206.67 ± 28.11 (64.88, 667.62) |

## S.5 Overall Results Grouped Data by Gender and Sample Time

Concentrations of the total particles and fluorescence tags used in the experiment, averaged by gender and grouped by sample time, are shown in Table S9, while their mean areas are shown in Table S10.

Table S9. Mean ± SE and range (minimum, maximum values in parentheses) results of particle concentration for different times for two gender groups, each containing 9 donors. FIM: first-in-morning sample, LM: late-morning samples, FIM+LM: daily average.

| Gender | Collection time | Identified Particles  (%) | Total particles  (10^3^ per mL) | Lipids  (10^3^ per mL) | Proteins  (10^3^ per mL) | Lipid-Proteins  (10^3^ per mL) | Calcium  (10^3^ per mL) | DNA  (per mL) |
| --- | --- | --- | --- | --- | --- | --- | --- | --- |
| Male | FIM | 35.05 ± 5.43 (14.37, 65.02) | 204.57 ± 18.44 (91.02, 369.03) | 10.91 ± 1.32 (3.43, 26.59) | 22.95 ± 2.87 (2.23, 55.27) | 26.61 ± 2.50 (5.85, 58.73) | 4.98 ± 0.97 (1.11, 22.66) | 132.03 ± 34.52 (28.04, 639.87) |
|  | LM | 44.35 ± 5.24 (17.62, 61.53) | 239.47 ± 37.06 (70.63, 799.16) | 27.74 ± 6.14 (1.64, 131.37) | 27.40 ± 5.43 (7.37, 136.05) | 43.86 ± 5.88 (12.66, 119.23) | 4.73 ± 0.94 (0.48, 16.18) | 101.84 ± 23.94 (9.78, 417.12) |
|  | FIM+LM combined | 39.70 ± 5.58 (14.37, 65.02) | 222.02 ± 29.28 (70.63, 799.16) | 19.33 ± 4.73 (1.64, 131.37) | 25.17 ± 4.33 (2.23, 136.05) | 35.23 ± 4.77 (5.85, 119.23) | 4.85 ± 0.95 (0.48, 22.66) | 116.93 ± 29.61 (9.78, 639.87) |
| Female | FIM | 51.48 ± 4.06 (25.35, 69.63) | 252.07 ± 51.60 (46.93, 1106.96) | 6.89 ± 0.98 (1.02, 20.19) | 39.16 ± 9.25 (5.14, 211.02) | 66.12 ± 8.71 (13.33, 217.55) | 3.45 ± 0.81 (0.41, 14.90) | 4104.77 ± 2018.10 (12.69, 36889.68) |
|  | LM | 55.68 ± 3.47 (35.38, 72.87) | 207.74 ± 30.03 (49.29, 529.55) | 9.16 ± 1.29 (1.69, 22.37) | 29.45 ± 4.85 (4.05, 100.84) | 73.08 ± 9.46 (17.25, 180.09) | 1.96 ± 0.46 (0.19, 10.03) | 1551.74 ± 781.45 (9.05, 16473.68) |
|  | FIM+LM combined | 53.58 ± 3.83 (25.35, 72.87) | 229.90 ± 42.13 (46.93, 1106.96) | 8.03 ± 1.16 (1.02, 22.37) | 34.31 ± 7.38 (4.05, 211.02) | 69.60 ± 9.03 (13.33, 217.55) | 2.70 ± 0.67 (0.19, 14.90) | 2828.26 ± 1541.61 (9.05, 36889.68) |

Table S10. Mean ± SE and range (minimum, maximum values in parentheses) results of particle mean area for different times for two gender groups, each containing 9 donors. FIM: first-in-morning sample, LM: late-morning samples, FIM+LM: daily average.

| Gender | Collection time | Total particles  (µm^2^) | Lipids  (µm^2^) | Proteins  (µm^2^) | Lipid-Proteins  (µm^2^) | Calcium  (µm^2^) | DNA  (µm^2^) |
| --- | --- | --- | --- | --- | --- | --- | --- |
| Male | FIM | 2.95 ± 0.24 (1.58, 4.79) | 6.85 ± 0.26 (5.30, 9.75) | 10.27 ± 0.64 (6.04, 5.92) | 8.82 ± 0.29 (7.00, 12.06) | 8.98 ± 0.55 (5.58, 14.88) | 165.35 ± 15.15 (74.21, 364.02) |
|  | LM | 3.35 ± 0.26 (1.36, 5.43) | 6.31 ± 0.25 (4.75, 9.02) | 9.03 ± 0.58 (4.87, 6.28) | 9.54 ± 0.40 (7.22, 6.65) | 8.11 ± 0.53 (5.39, 13.73) | 182.40 ± 8.78 (108.75, 253.26) |
|  | FIM+LM combined | 3.15 ± 0.25 (1.36, 5.43) | 6.58 ± 0.26 (4.75, 9.75) | 9.65 ± 0.62 (4.87, 16.28) | 9.18 ± 0.36 (7.00, 16.65) | 8.54 ± 0.54 (5.39, 14.88) | 173.88 ± 12.44 (74.21, 364.02) |
| Female | FIM | 5.35 ± 0.59 (2.52, 15.72) | 7.51 ± 0.39 (5.39, 12.02) | 12.03 ± 0.44 (8.46, 15.47) | 11.78 ± 0.78 (7.44, 22.15) | 10.43 ± 0.93 (5.22, 22.29) | 248.01 ± 42.10 (89.25, 667.62) |
|  | LM | 5.41 ± 0.39 (3.73, 9.80) | 7.30 ± 0.45 (5.37, 15.13) | 10.11 ± 0.61 (3.76, 16.26) | 12.48 ± 0.87 (7.83, 24.22) | 9.51 ± 0.99 (5.18, 20.64) | 230.92 ± 29.86 (64.88, 448.92) |
|  | FIM+LM combined | 5.38 ± 0.50 (2.52, 15.72) | 7.40 ± 0.42 (5.37, 15.13) | 11.07 ± 0.57 (3.76, 16.26) | 12.13 ± 0.82 (7.44, 24.22) | 9.97 ± 0.96 (5.18, 22.29) | 239.47 ± 36.21 (64.88, 667.62) |

## S.6 Overall Results Grouped Data by Age Group and Sample Time

Concentrations of the total particles and fluorescence tags used in the experiment, averaged by age group and grouped by sample time, are shown in Table S11, while their mean areas are shown in Table S12.

Table S11. Mean ± SE and range (minimum, maximum values in parentheses) results of particle concentration for different times for three age groups, each containing 6 donors. FIM: first-in-morning sample, LM: late-morning samples, FIM+LM: daily average.

| Age group  (years) | Collection time | Identified Particles  (%) | Total particles  (10^3^ per mL) | Lipids  (10^3^ per mL) | Proteins  (10^3^ per mL) | Lipid-Proteins  (10^3^ per mL) | Calcium  (10^3^ per mL) | DNA  (per mL) |
| --- | --- | --- | --- | --- | --- | --- | --- | --- |
| 25-29 | FIM | 50.47 ± 5.13 (21.44, 69.63) | 292.56 ± 61.54 (48.97, 1106.96) | 10.83 ± 1.52 (1.02, 24.12) | 46.68 ± 11.14 (9.04, 211.02) | 69.71 ± 10.46 (19.03, 217.55) | 3.41 ± 0.76 (0.44, 14.90) | 3661.13 ± 1717.04 (12.69, 25516.23) |
|  | LM | 57.58 ± 2.22 (48.64, 72.87) | 203.18 ± 30.44 (49.29, 420.99) | 25.36 ± 7.36 (1.69, 131.37) | 23.57 ± 3.64 (6.54, 80.93) | 66.57 ± 9.00 (17.25, 164.91) | 1.67 ± 0.27 (0.31, 4.16) | 1762.39 ± 951.08 (15.15, 16473.68) |
|  | FIM+LM combined | 54.03 ± 4.13 (21.44, 72.87) | 247.87 ± 48.96 (48.97, 1106.96) | 18.10 ± 5.45 (1.02, 131.37) | 35.12 ± 8.48 (6.54, 211.02) | 68.14 ± 9.62 (17.25, 217.55) | 2.54 ± 0.59 (0.31, 14.90) | 2711.76 ± 1384.02 (12.69, 25516.23) |
| 30-38 | FIM | 34.77 ± 4.67 (14.37, 55.41) | 200.69 ± 13.90 (91.85, 290.28) | 8.40 ± 1.13 (3.60, 26.59) | 20.64 ± 2.30 (2.23, 42.04) | 31.17 ± 3.48 (5.85, 68.09) | 5.00 ± 1.13 (0.71, 22.66) | 176.29 ± 45.38 (31.69, 818.62) |
|  | LM | 47.02 ± 4.85 (25.12, 72.16) | 250.51 ± 41.90 (74.79, 799.16) | 18.05 ± 3.28 (2.91, 62.10) | 35.56 ± 7.05 (4.05, 136.05) | 55.35 ± 8.47 (14.44, 180.09) | 3.56 ± 0.92 (0.19, 16.18) | 144.92 ± 28.53 (10.30, 417.12) |
|  | FIM+LM combined | 40.89 ± 5.25 (14.37, 72.16) | 225.60 ± 31.31 (74.79, 799.16) | 13.23 ± 2.62 (2.91, 62.10) | 28.10 ± 5.37 (2.23, 136.05) | 43.26 ± 6.79 (5.85, 180.09) | 4.28 ± 1.03 (0.19, 22.66) | 160.61 ± 37.52 (10.30, 818.62) |
| 53-65 | FIM | 44.55 ± 5.82 (17.87, 69.37) | 191.71 ± 18.95 (46.93, 333.35) | 7.48 ± 0.91 (1.27, 16.54) | 25.84 ± 2.56 (5.14, 55.27) | 38.20 ± 4.08 (11.25, 96.10) | 4.23 ± 0.78 (0.41, 14.34) | 2517.79 ± 1886.54 (27.30, 36889.68) |
|  | LM | 45.44 ± 5.77 (17.62, 67.12) | 217.13 ± 27.82 (64.93, 529.55) | 11.94 ± 1.61 (1.64, 29.75) | 26.15 ± 3.90 (5.72, 80.12) | 53.49 ± 7.57 (12.66, 159.00) | 4.80 ± 0.90 (0.63, 15.01) | 573.06 ± 195.27 (9.05, 3039.93) |
|  | FIM+LM combined | 45.00 ± 5.71 (17.62, 69.37) | 204.42 ± 23.65 (46.93, 529.55) | 9.71 ± 1.37 (1.27, 29.75) | 26.00 ± 3.25 (5.14, 80.12) | 45.85 ± 6.17 (11.25, 159.00) | 4.51 ± 0.83 (0.41, 15.01) | 1545.42 ± 1339.21 (9.05, 36889.68) |

Table S12. Mean ± SE and range (minimum, maximum values in parentheses) results of particle mean area for different times for three age groups, each containing 6 donors. FIM: first-in-morning sample, LM: late-morning samples, FIM+LM: daily average.

| Age group  (years) | Collection time | Total particles  (µm^2^) | Lipids  (µm^2^) | Proteins  (µm^2^) | Lipid-Proteins  (µm^2^) | Calcium  (µm^2^) | DNA  (µm^2^) |
| --- | --- | --- | --- | --- | --- | --- | --- |
| 25-29 | FIM | 4.39 ± 0.30 (2.59, 6.42) | 7.73 ± 0.35 (6.03, 11.65) | 11.91 ± 0.63 (6.04, 15.92) | 11.55 ± 0.95 (7.25, 22.15) | 9.11 ± 0.61 (5.22, 15.10) | 194.72 ± 30.96 (89.25, 667.62) |
|  | LM | 5.07 ± 0.48 (3.65, 9.80) | 7.04 ± 0.22 (5.93, 9.24) | 9.99 ± 0.59 (3.76, 16.26) | 11.89 ± 0.98 (7.49, 24.22) | 8.61 ± 0.94 (5.39, 20.64) | 183.17 ± 17.47 (64.88, 322.52) |
|  | FIM+LM combined | 4.73 ± 0.40 (2.59, 9.80) | 7.38 ± 0.30 (5.93, 11.65) | 10.95 ± 0.64 (3.76, 16.26) | 11.72 ± 0.96 (7.25, 24.22) | 8.86 ± 0.78 (5.22, 20.64) | 188.94 ± 24.81 (64.88, 667.62) |
| 30-38 | FIM | 3.48 ± 0.37 (1.58, 5.91) | 6.43 ± 0.17 (5.30, 7.72) | 10.07 ± 0.55 (6.41, 14.55) | 9.62 ± 0.46 (7.02, 14.90) | 8.62 ± 0.52 (5.58, 14.30) | 250.87 ± 41.19 (99.50, 650.69) |
|  | LM | 4.26 ± 0.42 (1.88, 6.72) | 6.13 ± 0.19 (4.96, 8.18) | 9.57 ± 0.69 (4.03, 16.28) | 11.56 ± 0.70 (7.82, 19.05) | 7.61 ± 0.36 (5.50, 10.11) | 232.98 ± 29.64 (88.38, 448.92) |
|  | FIM+LM combined | 3.87 ± 0.40 (1.58, 6.72) | 6.28 ± 0.18 (4.96, 8.18) | 9.82 ± 0.62 (4.03, 16.28) | 10.59 ± 0.61 (7.02, 19.05) | 8.11 ± 0.46 (5.50, 14.30) | 241.93 ± 35.43 (88.38, 650.69) |
| 53-65 | FIM | 4.59 ± 0.81 (1.65, 15.72) | 7.39 ± 0.40 (5.42, 12.02) | 11.48 ± 0.49 (7.95, 15.09) | 9.73 ± 0.34 (7.00, 12.68) | 11.39 ± 0.99 (6.64, 22.29) | 174.45 ± 23.03 (74.21, 504.69) |
|  | LM | 3.80 ± 0.32 (1.36, 5.43) | 7.25 ± 0.57 (4.75, 15.13) | 9.16 ± 0.53 (5.14, 14.81) | 9.58 ± 0.34 (7.22, 12.47) | 10.20 ± 0.88 (5.18, 19.32) | 203.83 ± 18.17 (100.08, 412.39) |
|  | FIM+LM combined | 4.20 ± 0.61 (1.36, 15.72) | 7.32 ± 0.48 (4.75, 15.13) | 10.32 ± 0.56 (5.14, 15.09) | 9.65 ± 0.34 (7.00, 12.68) | 10.80 ± 0.94 (5.18, 22.29) | 189.14 ± 20.76 (74.21, 504.69) |

## S.7 Overall Results Grouped Data by Gender and Age Group

Concentrations of the total particles and fluorescence tags used in the experiment, averaged by gender and grouped by age group, are shown in Table S13, while their mean areas are shown in Table S14.

Table S13. Mean ± SE and range (minimum, maximum values in parentheses) results of particle concentration for different genders for three age groups, each containing 3 donors. FIM: first-in-morning sample, LM: late-morning samples, FIM+LM: daily average.

| Gender | Age Group (years) | Identified Particles  (%) | Total particles  (10^3^ per mL) | Lipids  (10^3^ per mL) | Proteins  (10^3^ per mL) | Lipid-Proteins  (10^3^ per mL) | Calcium  (10^3^ per mL) | DNA  (per mL) |
| --- | --- | --- | --- | --- | --- | --- | --- | --- |
| Male | 25-29 | 50.06 ± 4.74 (21.44, 65.02) | 202.90 ± 25.57 (70.63, 420.99) | 27.76 ± 7.14 (3.46, 131.37) | 20.85 ± 2.40 (8.82, 52.29) | 47.96 ± 5.44 (19.03, 119.23) | 2.34 ± 0.31 (0.48, 6.66) | 131.49 ± 33.24 (15.15, 602.29) |
|  | 30-38 | 32.62 ± 3.90 (14.37, 48.21) | 254.72 ± 40.43 (74.79, 799.16) | 18.46 ± 3.32 (3.60, 62.10) | 29.83 ± 6.48 (2.23, 136.05) | 28.92 ± 5.04 (5.85, 116.22) | 6.48 ± 1.15 (1.26, 22.66) | 119.28 ± 25.97 (10.30, 417.12) |
|  | 53-65 | 36.42 ± 5.81 (17.62, 62.95) | 208.45 ± 17.13 (91.02, 365.96) | 11.77 ± 1.43 (1.64, 29.75) | 24.83 ± 2.99 (6.49, 55.27) | 28.82 ± 2.57 (11.25, 58.29) | 5.75 ± 0.97 (1.09, 15.01) | 100.03 ± 30.48 (9.78, 639.87) |
| Female | 25-29 | 57.99 ± 2.80 (43.92, 72.87) | 292.83 ± 63.69 (48.97, 1106.96) | 8.44 ± 1.43 (1.02, 20.68) | 49.39 ± 11.21 (6.54, 211.02) | 88.32 ± 11.41 (17.25, 217.55) | 2.74 ± 0.79 (0.31, 14.90) | 5292.03 ± 1808.65 (12.69, 25516.23) |
|  | 30-38 | 49.17 ± 4.56 (25.35, 72.16) | 196.48 ± 16.99 (91.94, 390.27) | 8.00 ± 0.83 (2.91, 16.98) | 26.37 ± 4.15 (4.05, 100.84) | 57.60 ± 7.35 (21.25, 180.09) | 2.08 ± 0.63 (0.19, 13.24) | 201.93 ± 45.29 (24.45, 818.62) |
|  | 53-65 | 53.57 ± 3.33 (37.44, 69.37) | 200.39 ± 29.26 (46.93, 529.55) | 7.64 ± 1.20 (1.27, 22.37) | 27.17 ± 3.57 (5.14, 80.12) | 62.87 ± 7.06 (13.33, 159.00) | 3.28 ± 0.60 (0.41, 10.03) | 2990.82 ± 1865.71 (9.05, 36889.68) |

Table S14. Mean ± SE and range (minimum, maximum values in parentheses) results of particle mean area for different genders for three age groups, each containing 3 donors. FIM: first-in-morning sample, LM: late-morning samples, FIM+LM: daily average.

| Gender | Age Group (years) | Total particles  (µm^2^) | Lipids  (µm^2^) | Proteins  (µm^2^) | Lipid-Proteins  (µm^2^) | Calcium  (µm^2^) | DNA  (µm^2^) |
| --- | --- | --- | --- | --- | --- | --- | --- |
| Male | 25-29 | 3.83 ± 0.17 (2.59, 4.79) | 7.27 ± 0.20 (5.93, 9.57) | 10.24 ± 0.61 (6.04, 15.92) | 9.37 ± 0.27 (7.25, 12.06) | 7.88 ± 0.37 (5.39, 10.85) | 193.40 ± 9.45 (90.31, 253.26) |
|  | 30-38 | 2.65 ± 0.17 (1.58, 3.92) | 5.95 ± 0.12 (4.96, 6.84) | 8.27 ± 0.53 (4.87, 16.28) | 9.17 ± 0.46 (7.02, 16.65) | 7.95 ± 0.51 (5.57, 14.30) | 170.52 ± 14.99 (111.67, 364.02) |
|  | 53-65 | 2.97 ± 0.29 (1.36, 5.43) | 6.52 ± 0.34 (4.75, 9.75) | 10.44 ± 0.64 (5.14, 14.81) | 8.99 ± 0.33 (7.00, 12.47) | 9.80 ± 0.63 (6.38, 14.88) | 157.70 ± 11.31 (74.21, 237.25) |
| Female | 25-29 | 5.62 ± 0.44 (3.99, 9.80) | 7.50 ± 0.38 (5.93, 11.65) | 11.66 ± 0.65 (3.76, 16.26) | 14.08 ± 1.15 (8.00, 24.22) | 9.84 ± 1.01 (5.22, 20.64) | 184.48 ± 34.29 (64.88, 667.62) |
|  | 30-38 | 5.09 ± 0.31 (2.52, 6.72) | 6.60 ± 0.20 (5.37, 8.18) | 11.36 ± 0.54 (4.03, 14.55) | 12.01 ± 0.62 (8.61, 19.05) | 8.27 ± 0.41 (5.50, 11.31) | 313.34 ± 41.53 (88.38, 650.69) |
|  | 53-65 | 5.43 ± 0.69 (3.73, 15.72) | 8.12 ± 0.55 (5.90, 15.13) | 10.20 ± 0.49 (6.73, 15.09) | 10.32 ± 0.29 (7.44, 12.68) | 11.80 ± 1.14 (5.18, 22.29) | 220.58 ± 25.23 (100.08, 504.69) |

## S.8 Statistical Analysis

p-value results for t-tests for time-of-day and gender comparisons, and ANOVA for age group analysis are shown in Table S15.

Table S15. p-values results for t-tests for time-of-day and gender comparisons, and ANOVA for age group analysis. The null hypothesis stated that there were no significant differences between the groups being compared.

| Main comparison | Subgroup | Variable of interest | p-value | | | | | | |
| --- | --- | --- | --- | --- | --- | --- | --- | --- | --- |
|  |  |  | Identified particles | All particles | Lipids | Proteins | Lipid-Proteins | Calcium | DNA |
| Time of day (t-test) | | Conc. | 0.010 | 0.945 | 0.004 | 0.700 | 0.085 | 0.280 | 0.194 |
|  |  | Area | - | 0.554 | 0.314 | 0.005 | 0.308 | 0.104 | 0.953 |
| Gender (t-test) | | Conc. | < 0.001 | 0.695 | 0.001 | 0.105 | < 0.001 | 0.008 | 0.005 |
|  |  | Area | - | < 0.001 | 0.013 | 0.008 | < 0.001 | 0.075 | 0.003 |
| Age group (ANOVA) | | Conc. | < 0.001 | 0.404 | 0.092 | 0.354 | 0.019 | 0.112 | 0.093 |
|  |  | Area | - | 0.175 | 0.006 | 0.213 | 0.026 | < 0.001 | 0.081 |
| Time of day (t-test) | Male | Conc. | 0.019 | 0.321 | 0.008 | 0.465 | 0.012 | 0.845 | 0.419 |
|  |  | Area | - | 0.137 | 0.086 | 0.118 | 0.133 | 0.175 | 0.227 |
|  | Female | Conc. | 0.083 | 0.436 | 0.133 | 0.379 | 0.434 | 0.099 | 0.189 |
|  |  | Area | - | 0.947 | 0.912 | 0.009 | 0.566 | 0.281 | 0.727 |
| Gender  (t-test) | FIM | Conc. | < 0.001 | < 0.001 | 0.016 | 0.098 | < 0.001 | 0.224 | 0.034 |
|  |  | Area | - | < 0.001 | 0.198 | 0.015 | < 0.001 | 0.172 | 0.032 |
|  | LM | Conc. | 0.001 | 0.526 | 0.004 | 0.687 | 0.009 | 0.008 | 0.045 |
|  |  | Area | - | < 0.001 | 0.036 | 0.145 | 0.003 | 0.263 | 0.057 |
| Time of day (t-test) | Younger | Conc. | 0.025 | 0.252 | 0.099 | 0.123 | 0.924 | 0.074 | 0.376 |
|  |  | Area | - | 0.214 | 0.225 | 0.068 | 0.879 | 0.342 | 0.829 |
|  | Middle | Conc. | 0.006 | 0.280 | 0.022 | 0.103 | 0.042 | 0.388 | 0.591 |
|  |  | Area | - | 0.137 | 0.266 | 0.616 | 0.052 | 0.130 | 0.719 |
|  | Older | Conc. | 0.861 | 0.467 | 0.045 | 0.956 | 0.163 | 0.672 | 0.349 |
|  |  | Area | - | 0.329 | 0.849 | 0.007 | 0.780 | 0.390 | 0.310 |
| Age group (ANOVA) | FIM | Conc. | 0.009 | 0.123 | 0.165 | 0.055 | 0.010 | 0.045 | 0.274 |
|  |  | Area | - | 0.260 | 0.039 | 0.097 | 0.094 | 0.017 | 0.217 |
|  | LM | Conc. | 0.009 | 0.704 | 0.182 | 0.406 | 0.512 | 0.055 | 0.135 |
|  |  | Area | - | 0.075 | 0.113 | 0.642 | 0.110 | 0.020 | 0.299 |
| Gender  (t-test) | Younger | Conc. | 0.051 | 0.137 | 0.044 | 0.029 | 0.010 | 0.543 | 0.007 |
|  |  | Area | - | < 0.001 | 0.933 | 0.112 | 0.001 | 0.291 | 0.734 |
|  | Middle | Conc. | < 0.001 | 0.205 | 0.012 | 0.710 | 0.014 | 0.006 | 0.152 |
|  |  | Area | - | < 0.001 | 0.013 | < 0.001 | 0.003 | 0.638 | 0.002 |
|  | Older | Conc. | < 0.001 | 0.818 | 0.064 | 0.679 | < 0.001 | 0.063 | 0.161 |
|  |  | Area | - | 0.001 | 0.024 | 0.786 | 0.014 | 0.144 | 0.025 |
| Age group (ANOVA) | Male | Conc. | < 0.001 | 0.417 | 0.114 | 0.481 | 0.029 | 0.012 | 0.788 |
|  |  | Area | - | < 0.001 | 0.002 | 0.044 | 0.816 | 0.019 | 0.105 |
|  | Female | Conc. | 0.058 | 0.113 | 0.780 | 0.061 | 0.083 | 0.535 | 0.064 |
|  |  | Area | - | 0.659 | 0.038 | 0.161 | 0.005 | 0.012 | 0.028 |

## S.9 Overall Results – Crystals

Concentrations and mean area of the crystal estimation methods defined in the experiment, for each participant separately, grouped by sample time, are shown in Table S16.

Table S16. Mean ± STD and range (minimum, maximum values in parentheses) results of particle concentration and mean areas of 3 different crystal estimation methods for different times for 18 donors individually. FIM: first-in-morning sample, LM: late-morning samples, FIM+LM: daily average.

| Donor | Time of sample | Birefringence by Area  Concentration  (particles/mL) | Birefringence by Calcein  Concentration  (particles/mL) | Only Birefringence  Concentration  (particles/mL) | Birefringence by Area  Mean Area (µm^2^) | Birefringence by Calcein  Mean Area (µm^2^) | Only Birefringence  Mean Area (µm^2^) |
| --- | --- | --- | --- | --- | --- | --- | --- |
| Female 1 | FIM | 11605.66 ± 4940.37 (6972.04, 16804.21) | 46.53 ± 12.07 (33.00, 56.19) | 892.51 ± 557.53 (525.62, 1534.10) | 38.54 ± 2.01 (36.55, 40.58) | 44.87 ± 13.43 (29.45, 54.00) | 6.23 ± 0.52 (5.66, 6.67) |
|  | LM | 5747.22 ± 2717.57 (3240.42, 8635.30) | 44.05 ± 13.53 (35.19, 59.62) | 110.35 ± 60.31 (41.49, 153.78) | 32.24 ± 1.75 (30.97, 34.24) | 33.44 ± 8.97 (23.44, 40.78) | 12.35 ± 6.51 (7.98, 19.83) |
| Female 2 | FIM | 1782.60 ± 1549.56 (574.38, 3529.63) | 34.32 ± 13.75 (23.55, 49.81) | 51.19 ± 51.43 (8.69, 108.35) | 36.42 ± 3.11 (34.02, 39.94) | 36.63 ± 3.88 (32.16, 39.17) | 18.06 ± 7.95 (11.03, 26.69) |
|  | LM | 1174.28 ± 450.77 (659.02, 1495.71) | 29.21 ± 12.34 (16.09, 40.58) | 17.74 ± 7.63 (13.05, 26.54) | 32.97 ± 2.04 (31.36, 35.26) | 38.98 ± 19.57 (24.06, 61.13) | 13.48 ± 0.86 (12.49, 14.01) |
| Female 3 | FIM | 2403.68 ± 757.02 (1856.02, 3267.53) | 62.65 ± 51.91 (12.08, 115.81) | 32.86 ± 35.47 (11.49, 73.81) | 37.59 ± 3.38 (33.70, 39.77) | 44.07 ± 2.51 (42.34, 46.95) | 21.38 ± 24.30 (3.93, 49.13) |
|  | LM | 1007.53 ± 181.87 (816.28, 1178.29) | 37.18 ± 18.16 (16.94, 52.05) | 10.75 ± 6.51 (6.59, 18.25) | 36.32 ± 1.72 (35.30, 38.31) | 38.50 ± 6.43 (32.70, 45.41) | 22.06 ± 14.86 (5.18, 33.20) |
| Female 4 | FIM | 1425.46 ± 859.94 (603.96, 2319.26) | 22.82 ± 16.58 (6.51, 39.65) | 105.18 ± 85.06 (33.44, 199.15) | 36.17 ± 1.42 (34.53, 37.05) | 43.12 ± 6.63 (37.54, 50.45) | 7.79 ± 2.33 (5.84, 10.38) |
|  | LM | 3558.22 ± 1475.65 (1901.24, 4730.72) | 48.17 ± 25.41 (30.82, 77.34) | 33.67 ± 24.87 (18.84, 62.38) | 35.08 ± 3.91 (30.75, 38.36) | 41.40 ± 9.14 (35.20, 51.89) | 10.09 ± 2.60 (8.39, 13.09) |
| Female 5 | FIM | 1302.18 ± 932.45 (708.08, 2376.89) | 28.33 ± 16.19 (12.71, 45.03) | 41.53 ± 25.40 (15.76, 66.55) | 39.80 ± 10.82 (33.17, 52.28) | 40.00 ± 17.64 (26.12, 59.85) | 12.73 ± 3.37 (9.19, 15.89) |
|  | LM | 1282.88 ± 245.41 (1050.59, 1539.59) | 104.84 ± 102.36 (39.81, 222.83) | 40.88 ± 29.92 (10.13, 69.89) | 33.04 ± 3.93 (28.86, 36.66) | 27.26 ± 6.18 (22.79, 34.31) | 10.61 ± 1.26 (9.33, 11.85) |
| Female 6 | FIM | 872.05 ± 314.68 (534.41, 1157.17) | 22.15 ± 15.17 (7.18, 37.52) | 189.64 ± 137.07 (95.45, 346.89) | 35.37 ± 3.51 (32.86, 39.38) | 41.23 ± 23.16 (16.16, 61.84) | 6.68 ± 2.00 (4.78, 8.77) |
|  | LM | 2028.09 ± 2726.62 (202.33, 5162.32) | 69.31 ± 72.98 (11.20, 151.21) | 31.60 ± 27.02 (2.53, 55.96) | 31.99 ± 4.01 (27.79, 35.78) | 28.22 ± 12.40 (14.70, 39.06) | 12.46 ± 4.03 (8.50, 16.55) |
| Female 7 | FIM | 2124.55 ± 599.95 (1524.86, 2724.76) | 40.03 ± 17.68 (25.10, 59.55) | 125.12 ± 99.27 (55.29, 238.75) | 39.05 ± 1.74 (37.19, 40.62) | 42.60 ± 7.78 (33.73, 48.25) | 13.50 ± 3.65 (9.44, 16.48) |
|  | LM | 4389.26 ± 5199.15 (881.01, 10362.43) | 67.31 ± 42.21 (25.33, 109.75) | 103.46 ± 158.54 (3.02, 286.24) | 36.37 ± 4.32 (32.54, 41.06) | 41.83 ± 7.01 (37.63, 49.92) | 11.64 ± 3.21 (8.51, 14.93) |
| Female 8 | FIM | 1099.09 ± 559.01 (482.56, 1572.90) | 40.25 ± 20.67 (17.32, 57.46) | 12.72 ± 2.44 (11.17, 15.53) | 34.55 ± 0.37 (34.15, 34.89) | 39.88 ± 4.41 (35.42, 44.24) | 22.79 ± 8.28 (16.05, 32.03) |
|  | LM | 1809.52 ± 525.80 (1450.73, 2413.08) | 81.96 ± 30.93 (50.20, 111.97) | 15.88 ± 4.07 (13.36, 20.57) | 38.77 ± 2.69 (36.70, 41.81) | 41.17 ± 9.41 (30.34, 47.34) | 13.73 ± 6.28 (9.64, 20.96) |
| Female 9 | FIM | 7783.68 ± 8208.55 (2808.64, 17258.11) | 46.28 ± 30.11 (26.86, 80.97) | 1837.44 ± 2393.42 (351.35, 4598.44) | 50.09 ± 16.02 (32.69, 64.25) | 38.60 ± 9.23 (32.69, 49.23) | 31.34 ± 13.28 (16.05, 39.96) |
|  | LM | 4047.92 ± 2417.54 (1531.60, 6352.78) | 48.10 ± 32.96 (14.39, 80.27) | 346.13 ± 365.92 (12.59, 737.54) | 36.96 ± 4.34 (32.60, 41.28) | 37.36 ± 3.64 (33.24, 40.12) | 6.53 ± 0.46 (6.06, 6.98) |
| Male 1 | FIM | 912.79 ± 357.67 (510.32, 1194.27) | 70.49 ± 55.40 (31.27, 133.87) | 9.98 ± 4.21 (6.83, 14.76) | 31.89 ± 3.57 (28.20, 35.33) | 23.50 ± 11.01 (16.22, 36.16) | 14.51 ± 10.76 (7.01, 26.84) |
|  | LM | 1289.54 ± 696.66 (524.38, 1887.13) | 43.94 ± 16.96 (25.21, 58.25) | 10.23 ± 1.95 (8.14, 11.99) | 36.85 ± 4.94 (32.22, 42.04) | 31.51 ± 6.00 (24.65, 35.82) | 16.10 ± 4.51 (10.93, 19.16) |
| Male 2 | FIM | 1346.63 ± 312.95 (1088.63, 1694.75) | 33.04 ± 13.24 (20.73, 47.03) | 254.58 ± 215.68 (6.20, 394.50) | 37.82 ± 2.80 (36.20, 41.05) | 41.12 ± 9.67 (32.77, 51.72) | 9.96 ± 3.76 (6.97, 14.19) |
|  | LM | 1460.75 ± 1203.24 (615.12, 2838.28) | 23.06 ± 34.66 (2.67, 63.08) | 191.82 ± 183.62 (21.38, 386.26) | 35.52 ± 2.03 (33.98, 37.81) | 32.49 ± 15.94 (14.56, 45.09) | 8.79 ± 4.51 (5.42, 13.91) |
| Male 3 | FIM | 1957.03 ± 998.25 (1264.93, 3101.36) | 44.06 ± 29.82 (18.90, 77.00) | 53.38 ± 39.68 (13.51, 92.87) | 35.91 ± 2.70 (32.95, 38.24) | 41.28 ± 4.70 (35.95, 44.85) | 10.62 ± 8.31 (4.55, 20.09) |
|  | LM | 854.54 ± 397.79 (537.39, 1300.87) | 25.86 ± 19.34 (9.39, 47.16) | 210.82 ± 145.11 (84.12, 369.13) | 36.39 ± 3.45 (33.55, 40.22) | 40.16 ± 13.93 (24.87, 52.12) | 7.78 ± 2.52 (5.38, 10.41) |
| Male 4 | FIM | 1115.40 ± 717.38 (385.98, 1820.09) | 44.49 ± 24.97 (15.81, 61.45) | 465.14 ± 399.33 (27.34, 809.39) | 36.05 ± 3.22 (32.34, 38.09) | 29.41 ± 3.34 (26.71, 33.16) | 4.94 ± 1.25 (3.52, 5.90) |
|  | LM | 1116.70 ± 1219.96 (156.61, 2489.48) | 248.92 ± 404.76 (5.05, 716.15) | 200.01 ± 147.77 (42.25, 335.21) | 39.00 ± 2.26 (36.95, 41.41) | 37.54 ± 3.85 (34.85, 41.95) | 7.16 ± 2.67 (4.08, 8.91) |
| Male 5 | FIM | 1031.52 ± 682.34 (304.38, 1657.85) | 51.34 ± 61.99 (3.91, 121.48) | 336.37 ± 94.73 (253.40, 439.59) | 40.80 ± 4.47 (35.65, 43.58) | 30.64 ± 16.03 (12.69, 43.55) | 10.27 ± 9.24 (4.80, 20.94) |
|  | LM | 542.14 ± 282.08 (231.87, 783.12) | 11.05 ± 8.90 (0.90, 17.52) | 174.79 ± 98.40 (72.45, 268.72) | 35.47 ± 3.96 (31.08, 38.77) | 29.86 ± 12.00 (16.00, 36.85) | 5.03 ± 0.68 (4.40, 5.75) |
| Male 6 | FIM | 816.22 ± 738.66 (201.81, 1635.77) | 46.33 ± 42.73 (3.96, 89.42) | 98.13 ± 104.69 (10.96, 214.24) | 39.38 ± 1.50 (37.65, 40.34) | 29.96 ± 7.67 (23.40, 38.39) | 9.12 ± 5.59 (2.69, 12.71) |
|  | LM | 1449.71 ± 1005.97 (534.51, 2526.81) | 44.32 ± 19.21 (33.23, 66.50) | 216.45 ± 108.15 (91.87, 286.26) | 32.78 ± 3.37 (30.66, 36.67) | 23.12 ± 4.98 (18.28, 28.23) | 5.16 ± 0.28 (4.93, 5.47) |
| Male 7 | FIM | 1641.30 ± 1940.48 (458.79, 3880.80) | 255.11 ± 403.28 (12.30, 720.63) | 5800.30 ± 9774.24 (116.87, 17086.52) | 33.09 ± 2.88 (31.28, 36.42) | 19.10 ± 6.11 (14.67, 26.07) | 4.07 ± 1.10 (2.89, 5.07) |
|  | LM | 1001.90 ± 419.43 (652.21, 1466.92) | 17.98 ± 9.88 (6.59, 24.31) | 462.56 ± 166.68 (325.44, 648.09) | 41.73 ± 3.63 (38.73, 45.77) | 38.59 ± 5.39 (33.02, 43.79) | 7.03 ± 1.89 (5.40, 9.10) |
| Male 8 | FIM | 769.64 ± 255.65 (495.73, 1001.91) | 26.02 ± 11.85 (16.40, 39.25) | 323.37 ± 252.94 (51.64, 551.98) | 41.31 ± 1.57 (39.62, 42.72) | 34.01 ± 6.32 (26.71, 37.90) | 6.67 ± 3.94 (3.91, 11.19) |
|  | LM | 1661.05 ± 1474.39 (652.10, 3353.11) | 50.34 ± 23.02 (30.76, 75.69) | 87.98 ± 101.51 (8.10, 202.21) | 37.76 ± 1.54 (36.16, 39.23) | 26.83 ± 7.40 (19.43, 34.23) | 13.96 ± 5.89 (7.17, 17.73) |
| Male 9 | FIM | 976.78 ± 587.59 (460.23, 1616.03) | 17.06 ± 4.84 (13.04, 22.44) | 65.22 ± 88.96 (9.09, 167.79) | 44.30 ± 5.45 (38.29, 48.92) | 26.54 ± 17.31 (12.03, 45.70) | 10.33 ± 4.16 (6.41, 14.70) |
|  | LM | 138575.67 ± 217483.13 (3348.82, 389448.99) | 2276.95 ± 3815.73 (52.66, 6682.91) | 997.49 ± 909.95 (153.20, 1961.29) | 43.30 ± 5.76 (36.65, 46.64) | 43.46 ± 12.23 (29.35, 50.78) | 13.36 ± 13.97 (4.88, 29.48) |
| Outlier 1 | FIM | 13137.85 ± 19125.39 (1605.77, 35214.59) | 73.05 ± 47.34 (38.55, 127.02) | 433.55 ± 692.38 (4.66, 1232.31) | 35.33 ± 3.23 (32.21, 38.66) | 38.88 ± 14.35 (22.79, 50.36) | 11.32 ± 7.12 (5.57, 19.28) |
|  | LM | 11605.66 ± 4940.37 (6972.04, 16804.21) | 46.53 ± 12.07 (33.00, 56.19) | 892.51 ± 557.53 (525.62, 1534.10) | 38.54 ± 2.01 (36.55, 40.58) | 44.87 ± 13.43 (29.45, 54.00) | 6.23 ± 0.52 (5.66, 6.67) |
