# Supplementary material for: Characterizing the particulate content of urine in healthy humans using flow cytometry
Source: PLoS One. 2025 May 22;20(5):e0324271. doi: 10.1371/journal.pone.0324271 (PMC12097575; doi:10.1371/journal.pone.0324271)
Supplement: Attachment — Submitted filename: Response_to_Reviewers_auresp_2.docx [file pone.0324271.s004.docx]

TO:

Tomasz W. Kaminski
Academic Editor
PLOS ONE

PONE-D-25-04538R1

Characterizing the particulate content of urine in healthy humans using flow cytometry

Dear Dr. Kaminski,

Thank you for the very positive appraisal of our manuscript.

As requested, we have now addressed the remaining point raised by Reviewer 2. We agree that it is a minor issue, but are glad to include this additional information which further clarifies our measurement protocol and the results. We reply in detail, below, to the Reviewer’s comment, and note where this information was included in the revised manuscript.

We have also reviewed the reference list to ensure that it is complete and correct, as submitted previously.

Best wishes,
Brian Berkowitz, on behalf of all co-authors

**Comments to the Author**
Reviewer #2: In the revised manuscript “Characterizing the particulate content of urine in healthy humans using flow cytometry” by Hirsch et al, previous concerns have been appropriately addressed. On further inspection, there is one important question that remains to be addressed:

1. How did the authors address the possible contribution of particles from the diluent (i.e. PBS) or staining reagents (e.g. any of the fluorescent stains used here)? Did running PBS alone (i.e. diluent without urine) reveal any particles that may have obscured / confounded conclusions of urine particle composition? Additionally, if there were any special requirements for diluent (whether additionally filtered or obtained from a particular vendor), this information should be provided to enhance reproducibility.

**RESPONSE:** A preliminary test was used to confirm that PBS and dye aggregates do not have a significant effect on the particle measurements. Potential background staining of particles originating from the PBS or dye aggregates was checked using the same labeling procedure that was performed on samples containing PBS (without additional filtering) instead of urine. The resulting particle concentration was less than 0.5% of the corresponding concentration in the urine samples in most cases, and no more than 2% in the highest case.

This information has now been included in the Revised Manuscript, at the end of Section 2.4.

As the effect of background staining was very low, we are not aware of any effect of the particular diluent on the final particle concentration or the relevance of a specific vendor.
